# Temporal expression patterns of fruit-specific *α- EXPANSINS* during cell expansion in bell pepper (*Capsicum annuum* L.)

**DOI:** 10.1186/s12870-020-02452-x

**Published:** 2020-05-28

**Authors:** Andrés Mayorga-Gómez, Savithri U. Nambeesan

**Affiliations:** Department of Horticulture, University of Georgia, 120 Carlton Street, Athens, GA 30602 USA

**Keywords:** Cell wall loosening, Gene expression, Fruit growth and development, Fruit ripening, Bell pepper

## Abstract

**Background:**

Expansins (EXPs) facilitate non-enzymatic cell wall loosening during several phases of plant growth and development including fruit growth, internode expansion, pollen tube growth, leaf and root development, and during abiotic stress responses. In this study, the spatial and temporal expression patterns of *C. annuum α- EXPANSIN* (*CaEXPA*) genes were characterized. Additionally, fruit-specific *CaEXPA* expression was correlated with the rate of cell expansion during bell pepper fruit development.

**Results:**

Spatial expression patterns revealed that *CaEXPA13* was up-regulated in vegetative tissues and flowers, with the most abundant expression in mature leaves. Expression of *CaEXPA4* was associated with stems and roots. *CaEXPA3* was expressed abundantly in flower at anthesis suggesting a role for CaEXPA3 in flower development. Temporal expression analysis revealed that 9 out of the 21 genes were highly expressed during fruit development. Of these, expression of six genes, *CaEXPA5*, *CaEXPA7*, *CaEXPA12*, *CaEXPA14 CaEXPA17* and *CaEXPA19* were abundant 7 to 21 days after anthesis (DAA), whereas *CaEXPA6* was strongly expressed between 14 and 28 DAA. Further, this study revealed that fruit growth and cell expansion occur throughout bell pepper development until ripening, with highest rates of fruit growth and cell expansion occurring between 7 and 14 DAA. The expression of *CaEXPA14* and *CaEXPA19* positively correlated with the rate of cell expansion, suggesting their role in post-mitotic cell expansion-mediated growth of the bell pepper fruit. In this study, a ripening specific *EXP* transcript, *CaEXPA9* was identified, suggesting its role in cell wall disassembly during ripening.

**Conclusions:**

This is the first genome-wide study of *CaEXPA* expression during fruit growth and development. Identification of fruit-specific EXPAs suggest their importance in facilitating cell expansion during growth and cell wall loosening during ripening in bell pepper. These *EXPA* genes could be important targets for future manipulation of fruit size and ripening characteristics.

## Background

Expansins are proteins that mediate cell wall loosening during cell enlargement mediated growth in plants [1, 2]. Additionally they are involved in cell wall modification during fruit softening, root growth, pollen tube growth and in abiotic stress responses [2–7]. Expansins belong to a large superfamily of genes which are mainly divided into four families based on sequence analysis; *α- EXPANSIN* (*EXPA*), *β- EXPANSIN* (*EXPB*), *EXPANSIN A-like* (*EXPLA*) and *EXPANSIN B-like* (*EXPLB*) [8, 9]. Of these, EXPA constitutes the largest family with well-documented roles in cell wall expansion [10] (Table 1). The EXPB family members also demonstrate cell wall loosening activity and this family includes group 1 grass pollen allergens which facilitate pollen tube invasion [9, 20, 21]. EXLA and EXLB proteins belong to smaller families and their biological function is not clearly understood [9].

**Table 1:** Classification and number of EXPANSINS in various species

Species	EXPA	EXPB	EXPLA	EXPLB	Total	Reference
*Capsicum annuum*	21	7	1	9	38	
*Nicotiana tabacum*	36	6	3	7	52	[11]
*Solanum lycopersicum*	25	8	1	4	38	[12]
*Solanum tuberosum*	24	5	1	6	36	[13]
*Arabidopsis thaliana*	26	6	3	1	36	[14]; TAIR
*Vitis vinifera*	20	4	1	4	29	[15]
*Malus* × *domestica*	34	1	2	4	41	[16]
*Glycine max*	49	9	2	15	75	[17]
*Populus trichocarpa*	27	3	2	4	36	[18]
*Oryza sativa*	33	18	4	1	56	[19]

EXPAs have been indicated to play a role in hypocotyl extension in cucumber [22, 23] and leaf initiation in tomato [24]. Expression of both *EXPAs* and *EXPBs* was associated with internode elongation in deep-water rice [20, 25, 26]. Certain members of the *EXPA* family regulate the initiation and elongation of root hairs in Arabidopsis, rice and soybean [5, 27, 28]. The expression of *EXPA* genes during fruit development suggests a role in fruit expansion and ripening in several fruit crops such as tomato, pear and peach [3, 4, 12, 29–31].

EXPAs typically consists of 250–275 amino acids and mainly contains three domains: an N-terminal 20–30 amino acid signal peptide; Domain 1, made of the double-psi beta-barrel (DPBB) domain which is structurally similar to the glycosyl hydrolase family 45 (GH45); and Domain 2, which has a β-sandwich fold and is categorized as the carbohydrate binding module family 63 (CBM63) [9, 21]. In spite of the similarity to GH45 enzymes, EXPs do not exhibit hydrolytic activity. In fact, EXPA mediates non-enzymatic cell wall loosening in a pH dependent manner to enable cell expansion [10, 32]. Although the precise mechanism of action is not fully elucidated, EXPs are involved in disrupting noncovalent bonds between cellulose microfibrils and xyloglucan [9, 10].

Fruit development, in many species including pepper (*C. annuum*), can be mainly divided into four phases: fruit set, cell division, cell expansion and ripening [33]. Among these, fruit growth primarily occurs during the cell division and cell expansion phases, with the vast majority of growth occurring during the latter phase. In tomato, post-mitotic cell expansion results in an overall increase in cell volume by over 30,000 fold [34]. In pepper, however, nearly all cell division occurs pre-anthesis and only post-mitotic cell expansion is noted post-anthesis [35, 36]. The potential roles of specific EXPs in regulating post-mitotic cell expansion and fruit growth in bell pepper have not been evaluated.

In this study, *EXPs* were identified from bell pepper and spatial expression patterns of *CaEXPA* transcripts in seedlings, root, stem, leaves and fruit were determined. In addition, temporal expression patterns of *CaEXPA* transcripts were determined during fruit growth and development. Finally, patterns of *CaEXPA* transcript abundance were associated with changes in the relative rate of cell expansion during fruit growth and development. This is the first genome-wide identification of *EXP* genes during fruit expansion and ripening in bell pepper.

## Results

### Characterization of *CaEXP* gene family

**Fig. 1:**
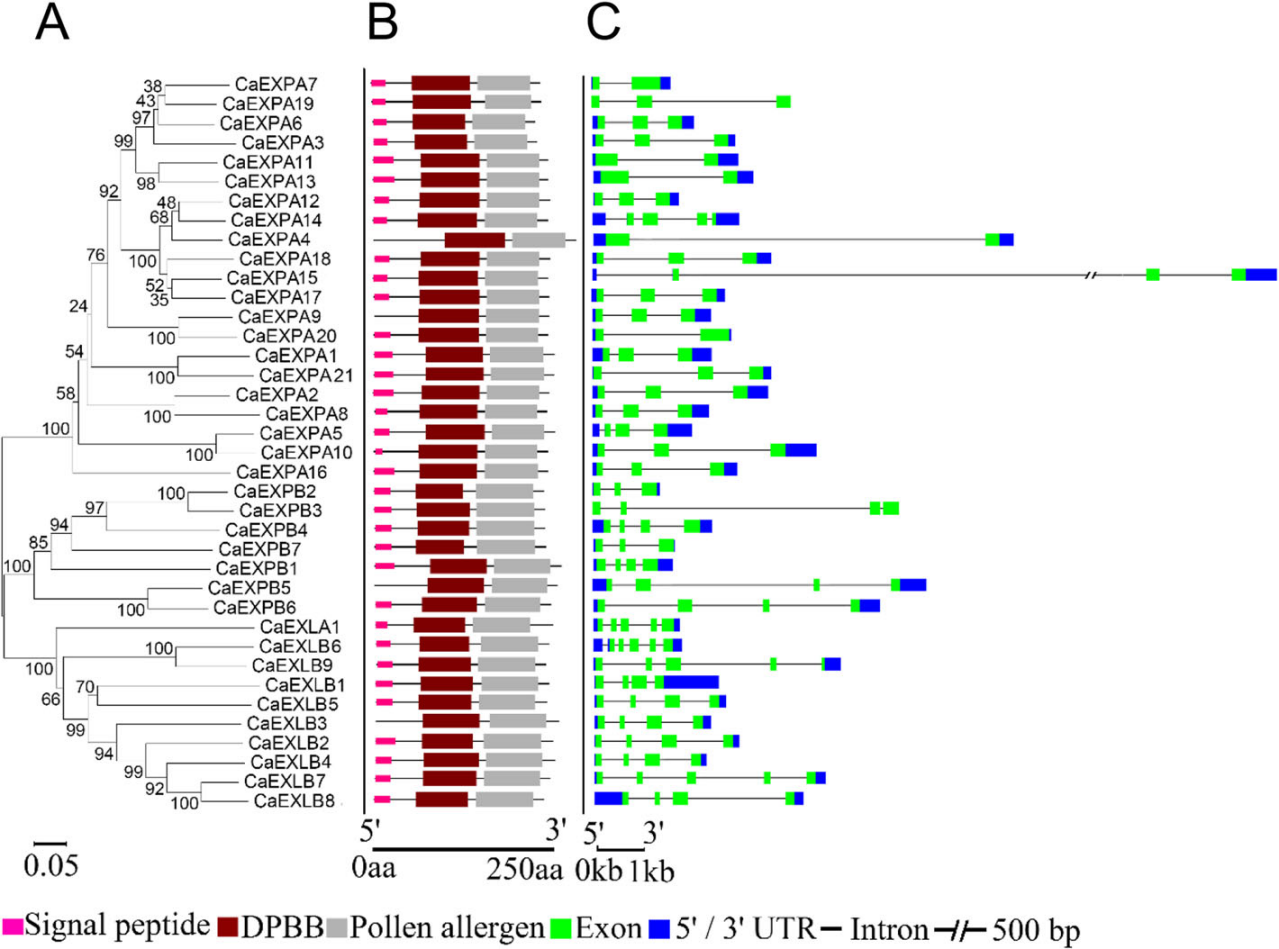
Phylogenetic analysis (**A**), schematic visualization of protein (**B**), and gene structure (**C**) of EXPANSINS in *C. annuum*. **A**. Phylogenetic tree performed using the neighbor joining method in MEGA 7.0 with 1000 bootstraps with p-distance model. **B**. Protein structure showing the signal peptide (pink), double-psi beta-barrel (DPBB; burgundy) and pollen allergen (grey) domains. Scale, 250 amino acids. **C**. Gene structure with exons in green, introns as a solid line and 5′ and 3′ UTR in blue. Break in second intron of *CaEXPA15* is 500 bp

A total of 40 *CaEXP* genes were identified from the *C. annuum* cv. Zunla-1 genome and sequences were retrieved from NCBI database [37] (Table 2). The length of the predicted protein sequences ranged from 239 to 298 amino acids (Table 2). Each EXP family has a characteristic sequence of conserved cysteine and tryptophan residues, and EXPA and EXPB also contain the conserved HFD motif [21]. Based on the conserved amino acids characteristic of a particular EXP family, 21 *CaEXPA*, 7 *CaEXPB*, 1 *CaEXLA* and 9 *CaEXLB* genes were identified (Table 2; Additional Fig. 1). All the conserved amino acids previously identified and described were present in CaEXPs with a few exceptions [21]. The characteristic HFD motif in EXPA and EXPB families was HFV in case of CaEXPA5 and CaEXPA10 and HLV in case of CaEXPB3 (Additional Fig. 1). While majority of the tryptophan residues were conserved in CaEXPA, they were not completely conserved in CaEXPB (Additional Fig. 1). Next, phylogenetic analysis was performed, which indicated that members were more closely related within families than between families (Fig. 1A). All the three domains, an N-terminal signal peptide domain, the double-psi beta-barrel (DPBB) domain and the CBM63 domain (homologous to group-2 pollen allergens in grass) were present in all the CaEXPs with the exception that the signal peptide was absent in CaEXPA4, CaEXPA9, CaEXPB5 and CaEXLB3 (Fig. 1B). Gene structure analysis indicated that *CaEXPs* had 1–5 introns (Fig. 1C). Most of the *CaEXPA* family members contained 1–2 introns with a few exceptions; *CaEXPA15* and *CaEXPA5* had 3 introns and *CaEXPA14* had 4 introns (Fig. 1C). All *CaEXPB* contained 3 introns except Ca*EXPB2* and *CaEXPB7* which had 2 introns each. *CaEXLA1* had 4 introns and *CaEXLB* introns were variable ranging from 2 to 5 (Fig. 1C).

**Table 2:** Nomenclature of *CaEXPs* with chromosome (Chr) position and amino acid length

NCBI Reference	Start position	End position	Length of aa	Chr	Gene Name
XM_016697484.1	6,719,269	6,721,107	265	1	*CaEXPA1*
XM_016698864.1	34,381,193	34,384,169	259	1	*CaEXPA2*
XM_016725129.1	128,210,443	128,229,587	241	1	*CaEXPA3*
XM_016704650.1	141,996,220	141,999,652	298	2	*CaEXPA4*
XM_016704699.1	142,745,201	142,754,239	266	2	*CaEXPA5*
NM_001324793.1	159,594,986	159,597,115	239	2	*CaEXPA6*
XM_016707763.1	12,037,829	12,040,394	249	3	*CaEXPA7*
XM_016707805.1	12,599,557	12,601,253	254	3	*CaEXPA8*
NM_001324929.1	1,850,043	1,853,156	256	4	*CaEXPA9*
XM_016714938.1	205,362,103	205,365,240	256	4	*CaEXPA10*
XM_016719265.1	198,682,908	198,687,183	258	5	*CaEXPA11*
XM_016722288.1	6,039,531	6,042,076	259	6	*CaEXPA12*
XM_016721684.1	138,349,371	138,352,444	258	6	*CaEXPA13*
XM_016720362.1	194,769,127	194,772,961	257	6	*CaEXPA14*
XM_016724770.1	105,746,513	105,750,286	258	7	*CaEXPA15*
XM_016724051.1	216,012,510	216,015,368	257	7	*CaEXPA16*
XM_016686945.1	210,752,505	210,757,330	259	9	*CaEXPA17*
XM_016687787.1	173,800,676	173,802,860	258	10	*CaEXPA18*
XM_016692780.1	197,808,765	197,811,908	249	11	*CaEXPA19*
XM_016694169.1	14,028,294	14,030,798	257	12	*CaEXPA20*
XM_016697490.1/XM_016697491.1	3209	7051	266	Unplaced	*CaEXPA21*
XM_016702157.1	229,210,413	229,212,116	275	1	*CaEXPB1*
XM_016702797.1	128,353,249	128,354,680	262	2	*CaEXPB2*
XM_016702801.1	128,491,704	128,498,302	269	2	*CaEXPB3*
XM_016719262.1	197,346,396	197,348,955	264	5	*CaEXPB4*
XM_016725274.1	196,431,721	196,438,912	269	7	*CaEXPB5*
XM_016689117.1 / XR_001666564.1	10,571,649	10,577,821	259	10	*CaEXPB6*
XM_016689293.1	172,669,446	172,671,192	257	10	*CaEXPB7*
XM_016684533.1	152,306,708	152,308,564	261	8	*CaEXLA1*
XM_016715121.1	14,318,386	14,321,060	255	1	*CaEXLB1*
XM_016698173.1	14,323,996	14,327,102	250	1	*CaEXLB2*
XM_016715143.1	14,381,013	14,383,514	251	1	*CaEXLB3*
XM_016698182.1	14,393,234	14,395,650	250	1	*CaEXLB4*
XM_016715155.1	14,401,512	14,404,354	252	1	*CaEXLB5*
XM_016698223.1 / XM_016698230.1	15,548,055	15,549,963	255	1	*CaEXLB6*
XM_016695791.1	298,511,555	298,516,534	252	1	*CaEXLB7*
XM_016721597.1	40,422,100	40,426,589	250	4	*CaEXLB8*
XM_016719579.1	40,488,989	40,494,318	249	4	*CaEXLB9*

### Spatial and temporal transcript abundance patterns of *CaEXPA* genes

**Fig. 2:**
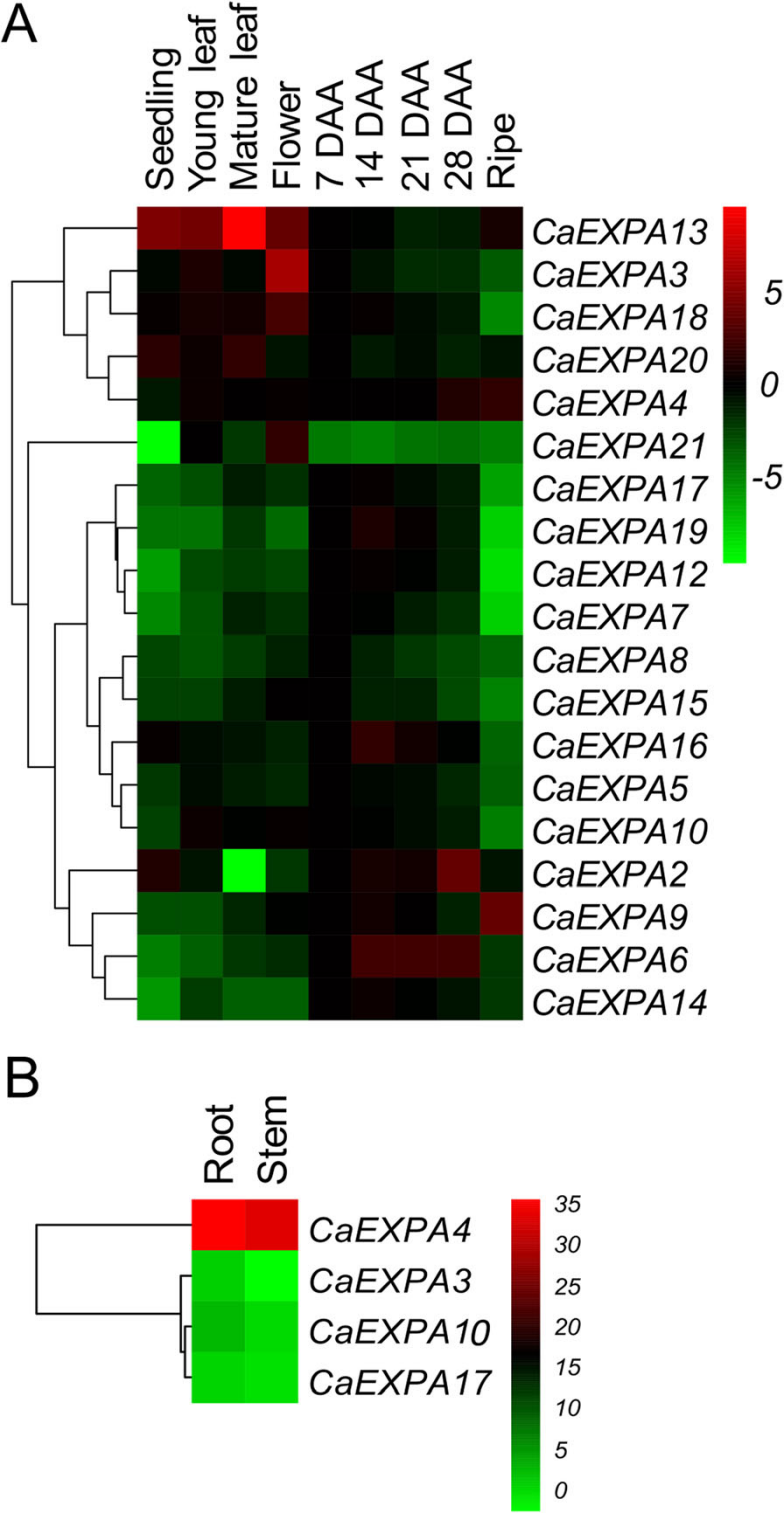
Spatio-temporal expression patterns of *CaEXPAs*. **A**. Expression patterns of 19 of the 21 *CaEXPA* genes. Transcript abundance of each *CaEXPA* gene in every tissue and fruit developmental time-point was normalized to its expression at 7 DAA, except *CaEXPA21*, which was normalized to its expression in young leaf. Transcripts of *CaEXPA1* and *CaEXPA11* were not detectable by qRT-PCR. **B**. Expression patterns of *CaEXPAs* in root and stem. Only four out of the 21 *CaEXPA* genes were expressed in root and stem tissue. Transcript abundance of all 4 genes were normalized to that of *CaEXPA10* in root. Tissues are displayed vertically and *CaEXPA* genes horizontally. Color scale from green to red indicates lower and higher transcript abundance, respectively. Expression is presented in a Log_2_ scale

The transcript abundance patterns of 21 *CaEXPA* genes was determined in seedlings, young and mature leaves, flowers at anthesis, and fruits at 7, 14, 21, 28 DAA and ripe stage (Fig. 2A). Of the 21 *CaEXPA* genes, 19 displayed expression in the tissues analyzed. The transcripts of *CaEXPA1* and *CaEXPA11* were not detectable. Among the 19 detectable transcripts, *CaEXPA13* showed highest expression in mature leaves and was generally higher in vegetative tissues and flowers in comparison to the fruit (Fig. 2A). *CaEXPA3, CaEXPA13*, *CaEXPA18* and *CaEXPA21* displayed highest transcript abundance in the flowers when compared to other tissues (Fig. 2A). In the case of fruit tissues for example, *CaEXPA17*, *CaEXPA19*, *CaEXPA12*, *CaEXPA7,* and *CaEXPA10* showed higher transcript abundance from 7 to 28 DAA and reduced abundance in ripe fruit (Fig. 2A). On the other hand, *CaEXPA9* and *CaEXPA4* transcript abundance was highest at the ripe fruit stage (Fig. 2A). Only 4 of the 21 *CaEXPA* genes, *CaEXPA3*, *CaEXPA4*, *CaEXPA10* and *CaEXPA17* were expressed in root and stem tissues (Fig. 2B). Of these, *CaEXPA4* showed a substantially higher expression compared to the other genes (Fig. 2B).

### Temporal transcript abundance patterns of *CaEXPAs* during fruit growth and development

**Fig. 3:**
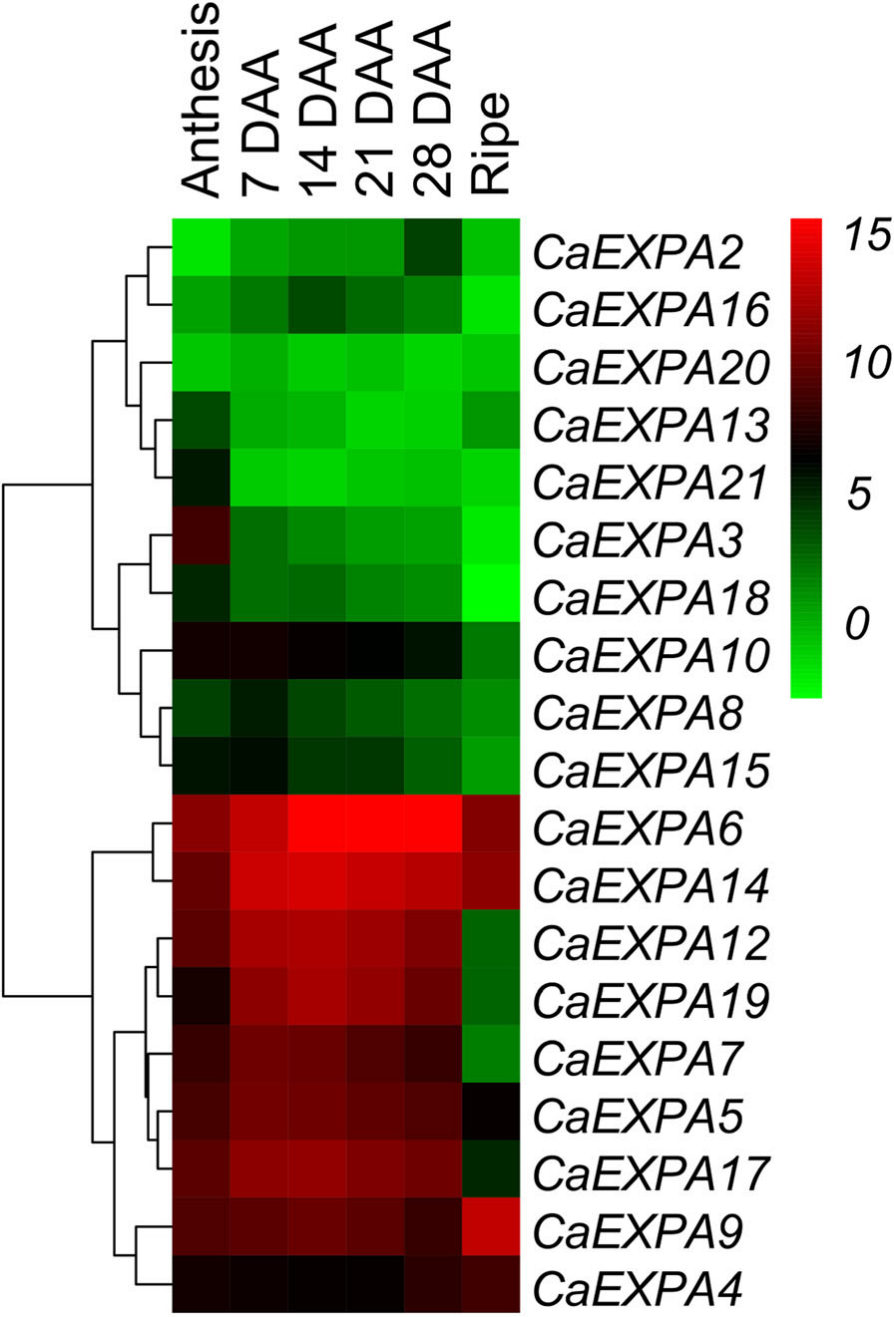
Temporal expression patterns of *CaEXPAs* during flower and fruit development. Transcript abundance of all *CaEXPA* genes was normalized to that of *CaEXPA21* at 7 DAA. Tissues are displayed vertically and *CaEXPA* genes horizontally. Color scale from green to red indicates lower and higher transcript abundance, respectively. Expression is presented in a Log_2_ scale

**Fig. 4:**
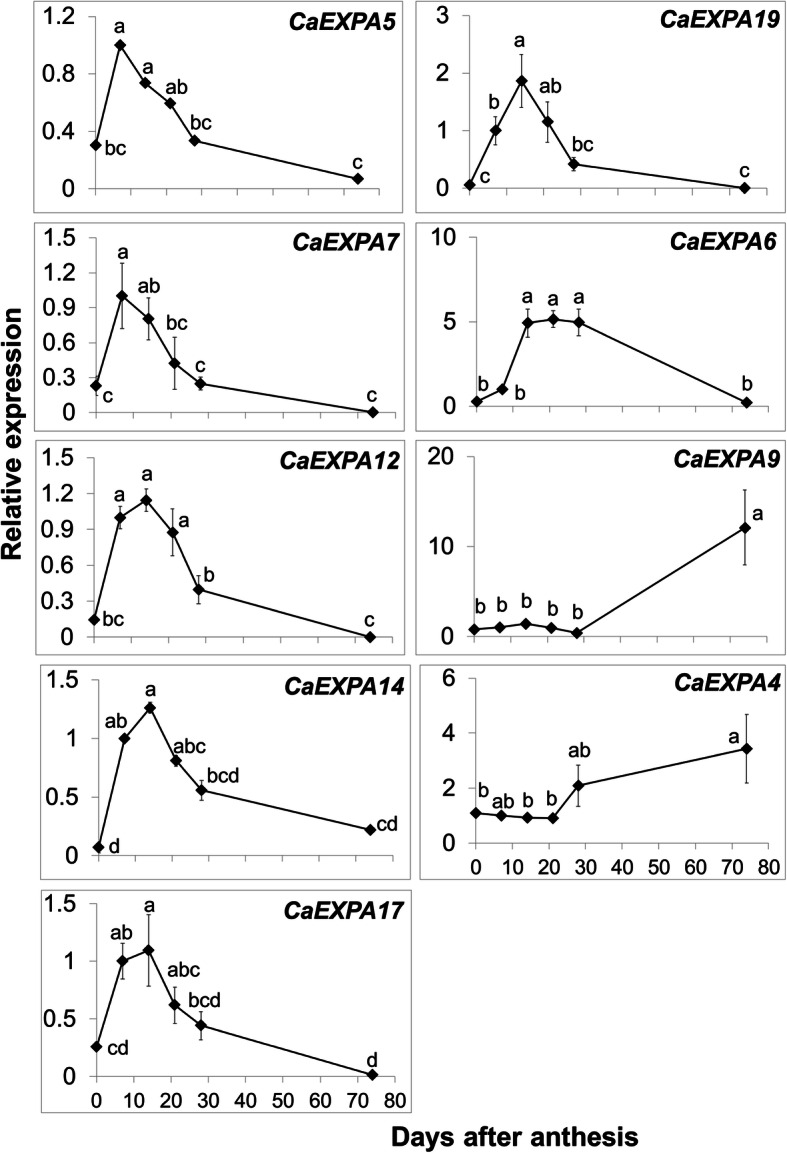
Relative transcript abundance of nine *CaEXPA* genes in flower and fruit development. These nine *CaEXPA* genes displayed relatively higher transcript abundance in Fig. 3. Values are means and standard errors of at least three replicates. Means separation was performed using Tukey’s HSD following test of significance using ANOVA (α = 0.05). Means followed by a different letter are significantly different

To identify the abundantly expressed *CaEXPA* transcripts within the fruit samples, transcript abundance levels of all *CaEXPAs* in flower and fruit tissues was normalized to that of *CaEXPA21* at 7 DAA (Fig. 3). Initially, clustering of the transcript abundance data separated *CaEXPA* genes into 2 groups, one set with nine genes showing higher expression compared to the other set, with 10 genes displaying lower expression during fruit development (Fig. 3). Of the latter 10 genes, *CaEXPA3* was highly expressed at anthesis (Fig. 3; Additional Fig. 2). Of the remaining nine highly abundant transcripts, several patterns of *EXPA* expression were evident during fruit development. The expression of *CaEXPA5*, *CaEXPA7, CaEXPA12*, *CaEXPA14* and *CaEXPA17* increased significantly from anthesis to 7 DAA and remained high until 21 DAA after which it gradually decreased; the expression of these five genes were positively correlated to each other (Figs. 3 and 4; Table 3). The expression of *CaEXPA19* similarly increased significantly from anthesis to 7 DAA, however the peak expression was at 14 DAA after which it gradually decreased and positively correlated with *CaEXPA12*, *CaEXPA14* and *CaEXPA17*. *CaEXPA6* expression was high from 14 to 28 DAA (Figs. 3 and 4; Table 3). *CaEXPA9* transcript abundance was high only in ripe fruit and increased by 13.2-fold in ripe fruit as compared with previous stages (anthesis until 28 DAA; Fig. 4). *CaEXPA4* as such was not substantially altered during early fruit development, however, its transcript abundance increased by 3.8-fold from 28 DAA until ripening and was negatively correlated with that of *CaEXPA5*, *CaEXPA12*, *CaEXPA14* and *CaEXPA17* (Fig. 4; Table 3).

**Table 3:**
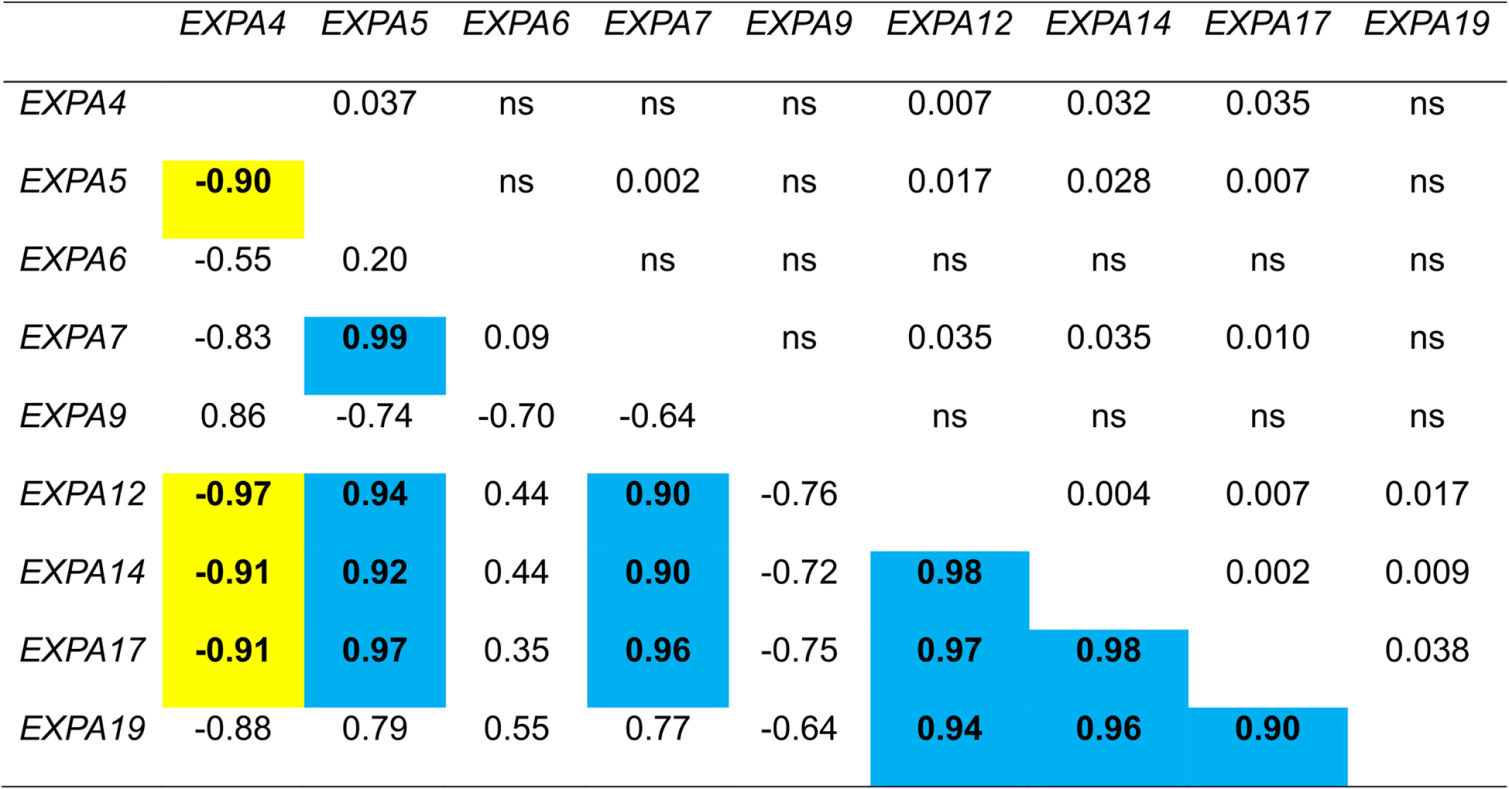
Correlation among abundantly expressed fruit *C. annuum EXPA* genes

Shown below the diagonal are correlations, significant positive correlation (blue) and significant negative correlation (yellow). Shown above the diagonal are *P* values, ns denotes not significant

### Fruit growth patterns in bell pepper

**Fig. 5:**
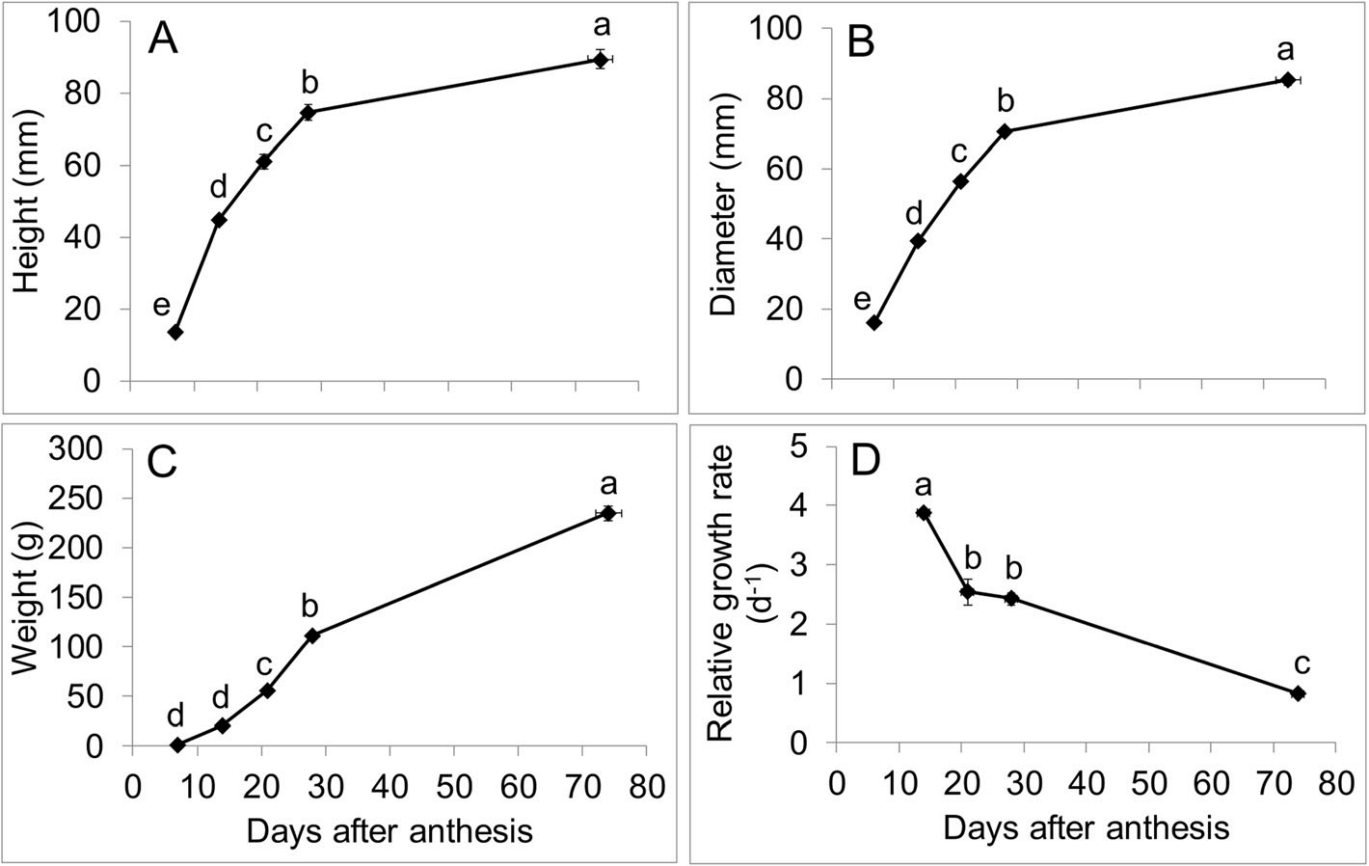
**A**. Fruit height, **B**. Fruit diameter, **C**. Fruit weight at five time-points during fruit development at various days after anthesis (DAA): 7, 14, 21, 28 DAA and ripe stage. **D**. Relative growth rate calculated using fruit weight (d^−1^) at four different intervals during fruit growth and development: from 7-14 DAA, 14–21 DAA, 21–28 DAA and 28 DAA to the ripe stage. Means separation was performed using Tukey’s HSD following test of significance using ANOVA (α = 0.05). Means followed by a different letter are significantly different

Fruit height increased by 3.3-fold between 7 and 14 DAA. It subsequently increased by 1.4-, 1.2- and 1.2-fold between 14 and 21 DAA, 21–28 DAA and 28 DAA-ripe, respectively (Fig. 5A). Increase in fruit diameter was 2.4-fold between 7 and 14 DAA and fold-changes across other developmental time-points were similar to that in fruit height (Fig. 5B). Fruit weight increased by 14.7-fold between 7 and 14 DAA. Subsequently, it increased by 2.7-, 2- and 2.1-fold between 14 and 21 DAA, 21–28 DAA and 28 DAA-ripe, respectively (Fig. 5C). Relative fruit growth was the highest between 7 and 14 DAA; it was 1.5-, 1.6- and 4.8-fold higher in comparison to that between 14 and 21 DAA, 21–28 DAA, and 28 DAA-ripe, respectively (Fig. 5D).

### Cell expansion rate during fruit development and correlation with *CaEXPA* transcript abundance

**Fig. 6:**
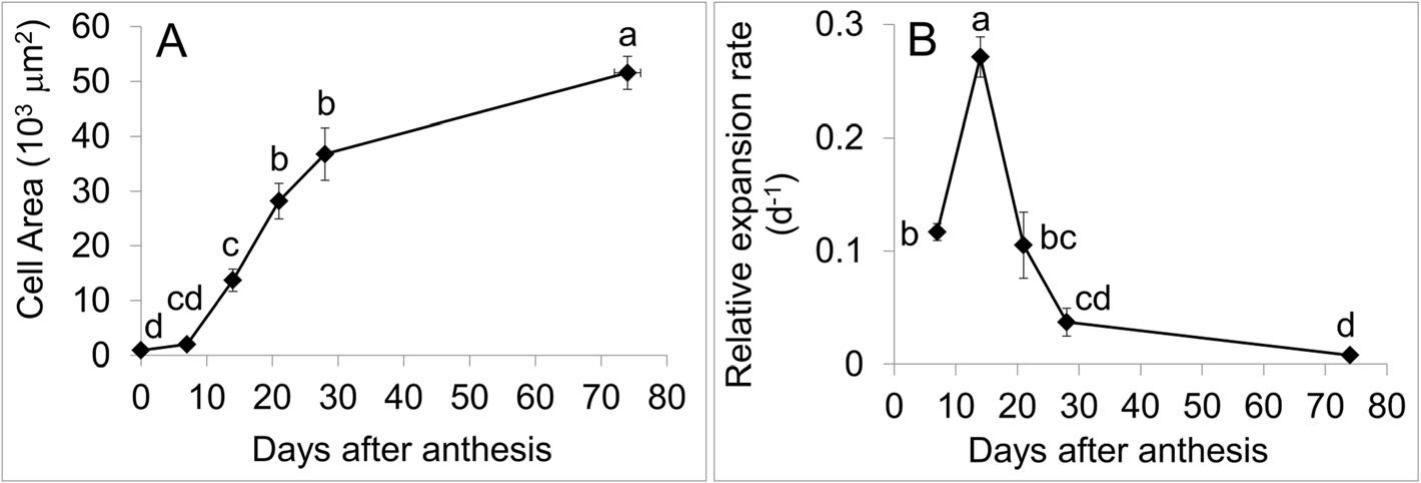
**A**. Fruit cell area (× 10^3^ μm^2^) at various stages during fruit development: 0, 7, 14, 21, 28 DAA and ripening. **B**. Relative fruit cell expansion rate (d^− 1^) from 0 to 7 DAA, 7–14 DAA, 14–21 DAA, 21–28 DAA and 28 DAA to the ripe stage. Means separation was performed using Tukey’s HSD following test of significance using ANOVA (α = 0.05). Means followed by a different letter are significantly different

Cell area increased gradually during fruit development, particularly between 7 and 28 DAA. Over the course of development, fruit pericarp cell area increased by around 60-fold (Fig. 6A). The relative cell expansion rate was highest between 7 and 14 DAA and subsequently declined over the rest of fruit development (Fig. 6B).

Correlations between the transcript abundance and the relative cell expansion rate during fruit growth were performed. Among the highly expressed *EXPA* genes in the fruit, only the transcript abundance of *CaEXPA14* and *CaEXPA19* significantly correlated with the relative cell expansion rate (Table 4).

**Table 4:** Correlation among abundantly expressed fruit *CaEXPA* genes and cell expansion rate. Significant positive correlations are in bold and ns denotes not significant

*CaEXPs*	Correlation to cell expansion rate	*P* value
*CaEXPA4*	−0.74	ns
*CaEXPA5*	0.72	ns
*CaEXPA6*	0.44	ns
*CaEXPA7*	0.74	ns
*CaEXPA9*	−0.49	ns
*CaEXPA12*	0.86	ns
*CaEXPA14*	**0.93**	0.023
*CaEXPA17*	0.86	ns
*CaEXPA19*	**0.97**	0.008

## Discussion

The *CaEXPA* family was the most abundant among the *EXPs* in bell pepper, similar to that observed in other *Solanaceae* crops such as tobacco (36 *EXPAs*) [11], tomato (25 *EXPAs*, Additional Fig. 3) [12], and potato (24 *EXPAs*) [13]. This is also true for other species including Arabidopsis, grape, apple, soybean, poplar and rice in which the largest number of *EXP* genes belong to the *EXPA* family (Table 1) [14–19]. CaEXPs contained 239 to 298 amino acid residues, similar to what was observed in tobacco and tomato [11, 12]. Classification of the CaEXP family was based on identification of conserved amino acids and characteristic domains within each family [21]. Further, the grouping of CaEXP families was confirmed in the phylogenetic analysis. The inconsistencies in the occurrence of the conserved tryptophan residues in CaEXPBs and lack of conservation of the HDF motif in certain CaEXPAs in bell pepper were also noted in EXP gene families from tomato, potato and grape [12, 15, 38]. The differences in conserved amino acid residues may suggest differences in substrate specificity and cell wall loosening activity [12]. The protein domains and exon-intron structure of CaEXP family were similar to other species such as sugarcane [39] and tobacco [11].

The spatial transcript abundance patterns revealed that *CaEXPA13* is the most highly expressed *EXPA* in vegetative tissues such as seedlings, young and mature leaves compared with fruit, suggesting a role in leaf development. Indeed, a transgenic approach has shown that certain EXPAs play a role during leaf initiation and development in *Arabidopsis* [40] and tobacco [41]. In this study, in comparison with *CaEXPA13*, *CaEXPA18* and *CaEXPA21*, the transcript abundance of *CaEXPA3* was greater in flowers compared to the other tissues and therefore CaEXPA3 may play a role in flower development. In several other studies, expression of *EXP* has been associated with pollen, stigma, and ovary where it may facilitate pollen tube growth by loosening of stigma cell walls [2, 42, 43]. Further *EXPAs* have also been shown to regulate petal development in petunia and rose [44, 45]. However, since the entire flower tissue was pooled together in this study, floral organ-specific expression patterns of *CaEXPA3* could not be determined. In this study, only 4 out of 21 *EXPA* genes were expressed in the stem and root tissues, among which *CaEXPA4* showed significantly higher expression. Hence it is likely that CaEXPA4 is associated with stem elongation and root growth. The role of certain members of EXP in stem elongation has been well established in rice [20] and root development in Arabidopsis [5].

Fruit growth is one of the longest phases of development. In tomato, although fruit growth does not increase significantly after the mature green stage of development, cell expansion within the pericarp tissue may continue until ripening [46, 47]. The highest rate in cell expansion occurred at 4 DAA in tomato [46]. Our results indicated that in bell pepper, fruit growth and cell expansion occur throughout fruit development until ripening. More frequent sampling during later stages of fruit development is necessary to confirm if these processes continue during ripening. The highest rates of fruit growth and cell expansion occurred during early fruit growth, particularly between 7 and 14 DAA, as clearly evident from the analysis of relative rates of fruit growth and cell expansion. These data suggest that increase in fruit growth during this period is largely facilitated by cell expansion.

Post-mitotic cell expansion-mediated growth is largely driven by the cell turgor pressure, associated with water-uptake, often into the cell vacuoles. During turgor-mediated growth, cell wall stress relaxation occur which results in cell wall loosening [48]. Expansins have been shown to induce cell wall loosening to promote cell wall enlargement by disrupting noncovalent bonds between cellulose microfibrils and xyloglucan [9, 10]. In this study, analysis of temporal transcript abundance patterns revealed higher expression of nine *CaEXPA* genes in the fruit. These genes displayed varying patterns associated with distinct fruit developmental stages. Although the expression of six genes, *CaEXPA5*, *CaEXPA7*, *CaEXPA12*, *CaEXPA14 CaEXPA17* and *CaEXPA19* increased during early stages of fruit growth, only the transcript abundance of *CaEXPA14* and *CaEXPA19* were significantly and positively correlated with the relative rate of cell expansion. These data indicate that at least *CaEXPA14* and *CaEXPA19* are associated with post-mitotic cell expansion-mediated growth of the bell pepper fruit. In addition, the transcript abundance patterns of *CaEXPA14* and *CaEXPA19* were strongly and significantly correlated with *CaEXPA5, CaEXPA7, CaEXPA12* and *CaEXPA17*, all of which are upregulated during early stages of fruit growth suggesting they may as well be associated with cell expansion during fruit growth. The transcript abundance of *CaEXPA6* was highest between 14 and 28 DAA and therefore CaEXPA6 may facilitate cell expansion during later stages of fruit development. These data indicate that several EXPAs may be important in mediating cell expansion during bell pepper fruit growth. Similarly, the expression of several *EXP* genes were associated with the expansion of immature fruit in tomato and tubers in potato [12, 13, 29, 38].

Ripening is the final stage of fruit development where multiple textural changes and fruit softening occur, mediated in part by EXPs [29, 33]. Ripening-specific *EXPs* have been identified in several fruits such as tomato, banana, pear and strawberry [3, 31, 49, 50]. Among the *EXPAs* analyzed in this study, *CaEXPA4* and *CaEXPA9* displayed high transcript abundance during late stages of fruit development in bell pepper. These genes did not appear to have substantial changes in transcript abundance during early fruit development; *CaEXPA4* increased by 3.8-fold and *CaEXPA9* displayed a dramatic rise by over 12-fold from 21 DAA until ripening. Thus, it is likely that *CaEXPA9* plays a predominant role in fruit softening during ripening in bell pepper.

## Conclusions

This study presents the first genome-wide report of the spatio-temporal expression patterns of *EXPA* genes in bell pepper. Expression of seven *CaEXPA* genes, *CaEXPA5*, *CaEXPA6, CaEXPA7*, *CaEXPA12*, *CaEXPA14 CaEXPA17* and *CaEXPA19* was associated with cell expansion and fruit growth. These results suggest the role of specific *CaEXPA* genes in facilitating cell expansion during fruit growth. The phase of cell expansion during fruit development is important in contributing to final fruit size in bell pepper. Further, physiological disorders such as blossom-end rot, are initiated during the period of cell expansion, with higher cell expansion rates resulting in potentially greater susceptibility. Functional characterization of these *CaEXPA* genes may help evaluate their direct roles in regulation of fruit growth and blossom-end rot incidence. This study also revealed *CaEXPA9* to be a fruit ripening specific gene which may play a role in ripening associated fruit softening in bell pepper. Thus, *CaEXPA9* could be a potential target for slowing down cell wall disassembly during ripening and postharvest storage.

## Methods

### Plant material

*Capsicum annuum* cv. Aristotle, a sweet type pepper (Seminis, St. Louis, MO, USA) was grown in the greenhouse from Dec 2017-May 2018 in Athens, GA. Initially, seeds were germinated in plug nursery trays in a low temperature incubator (Model 2015; VWR International, Randor, PA, USA) at 25 °C with 12 h of light and dark. Subsequently the seedlings were transplanted in Fafard 3B Mix Metro-Mix 830 (Sun Gro Horticulture, Agawam, MA, USA) in a 11 L plastic nursery container. During the experiment, the minimum and maximum temperature in the greenhouse was set at 15 °C and 26 °C respectively, with relative humidity at 66%.

### Experimental design and tissue collection

A completely randomized design was used and plant tissue samples were harvested in four replicates. Tissues were harvested from seedlings, roots, stems, leaves, flowers, and fruit from five developmental stages. Seedlings (above ground tissue) were harvested 15 days after germination when approximately 4 true leaves were present. Roots, stems, and young and mature leaves were harvested separately from the same plant before flowering. Young leaves were defined as apical and expanding whereas mature leaves were basal and fully expanded. Stem tissue was harvested from the apical region by collecting approximately 3 internodes. Fully open flowers were harvested at anthesis. At anthesis, individual flowers were tagged, manually pollinated and fruit samples were collected at 7 days after anthesis (DAA), 14 DAA, 21 DAA, 28 DAA and at the ripe stage. Fruit was considered ripe when the fruit changed from green to completely red.

### Identification and nomenclature of the *CaEXP* gene families

All CaEXPA sequences were obtained from National Center for Biotechnology Information (NCBI) and SolGenomics databases. All sequences were compared between the two databases and within each database to eliminate identical sequences. The sequences of CaEXPB, CaEXPLA and CaEXPLB were obtained from NCBI database and were compared using Clustal X (http://www.clustal.org/) to determine unique sequences within each gene family. In case of three *CaEXP* genes, *CaEXPA21*, *CaEXPB6*, and *CaEXLB6*, two transcripts variants were detected in NCBI (Table 2); the longer transcript variant X1 was used for all the subsequent analysis. Nomenclature for all the *CaEXP* gene sequences within a gene family were in the order of the most recent publication date followed by the chromosomal location information from NCBI. Since all the *EXP* sequences were from *C. annuum* cv. Zunla-1 [37], genes were ordered based on their respective chromosomal position (Table 2).

### Phylogenetic analysis, protein domain identification and gene structure prediction

The CaEXP sequences were aligned using Clustal X (http://www.clustal.org/). The alignment was verified using the Muscle option on MEGA 7 [51]. The phylogenetic tree was generated using the neighbor joining method on MEGA 7 using p-distance model, partial deletion for missing data treatment and 1000 bootstrap replicates. To identify protein domains, the location of signal peptide, double psi beta-barrel DPBB_1, and Pollen allergen regions in protein sequences were obtained from the software SMART (http://smart.embl-heidelberg.de/) along with PFAM (https://pfam.xfam.org/). For gene structure predictions, the positions of exons, introns, 5′ UTR and 3′ UTR for CaEXPAs were obtained from the NCBI database. Subsequently, visualization of gene and protein structure was performed using GSDS 2.0 (http://gsds.cbi.pku.edu.cn/).

### Primer design

Primers for *CaEXPAs* and reference genes were designed using ApE- A Plasmid Editor (http://jorgensen.biology.utah.edu/wayned/ape/) software (Additional Table 1). Three reference genes, *UBIQUITIN-CONJUGATING ENZYME* (*UBE2*, NCBI accession: DQ924970)*, GLYCERALDEHYDE-3-PHOSPHATE DEHYDROGENASE*-(*GAPDH*, NCBI accession: XM_016722738.1)*, 18S RIBOSOMAL RNA* (*18S rRNA*, NCBI accession, EF564281.1) were developed to normalize the expression of *CaEXPA* genes [52]. Of these, two reference genes *UBE2* and *GAPDH* were used for seedling, leaf, flower, and fruit tissues, and *GAPDH* and *18S rRNA* for root and stem tissues. *18S rRNA* was used since expression of *UBE2* was not detectable in root and stem tissues. All primer pairs were checked for their specificity using NCBI primer-blast (https://www.ncbi.nlm.nih.gov/tools/primer-blast/).

### RNA extraction, cDNA synthesis, qRT-PCR and generation of heatmap

For gene expression analyses, a total of four replicates were used for all tissues. For each replicate, to ensure sufficient material for RNA extraction, variable numbers of organs were collected. One replicate consisted of four seedlings, 10 flowers, 10 fruit from 7 DAA, and four fruit for 14 DAA, 21 DAA, 28 DAA and ripe stages. For a single replicate, four young and mature leaves, entire roots, and stem section containing approximately 3 internodes were harvested from a single plant as mentioned previously. All tissue samples were immediately frozen in liquid N_2_ and stored at − 80 °C until further analysis. RNA from root, stem and fruit tissues was extracted using the protocol described in [53]. However, this protocol did not yield RNA for seedling, leaf and flower and therefore RNA from these tissues was isolated using the E.Z.N.A. Total RNA Kit I (Omega Bio Tek, Norcross, GA) following the “difficult samples protocol” outlined in the manufacturer’s instructions. In the final step, RNA was resuspended in 30–40 μl of diethyl pyrocarbonate (DEPC) - treated water. RNA quality was evaluated using Nanodrop (Thermo Scientific Nanodrop 8000 Spectrophotometer, Waltham, MA) and the 260/280 absorbance was between 2.0 to 2.1 for all samples. The integrity of RNA was visualized on a 1.2% agarose gel with 0.5X Tris-Borate-EDTA (EMD Chemicals Inc., Gibbstown, NJ, USA) buffer. cDNA was prepared from 1 μg of RNA following the manufacturers protocol (Promega, Madison, WI, USA) and diluted to 100 μl [53].

Forward and reverse primers (0.2 μM) and cDNA (1 μl) were combined with PowerUP SYBR Green PCR Master Mix (Applied Biosystems, Foster City, CA, USA) for qRT-PCR reactions using Stratagene MX 3005P qRT-PCR System (Agilent Technologies, CA) following: 50 °C for 2 min, 95 °C for 5 min, followed by 95 °C for 15 s, and 60 °C for 1 min repeated for 40 cycles. This was followed by a melting curve analysis, 95 °C for 1 min, 55 °C for 30 s, 95 °C for 30 s. LinRegPCR (v. 11.0) was used to determine PCR reaction efficiency. Relative gene expression was determined following efficiency correction according to [53]. To generate the heatmap, data were transformed using: Log_2_ (X + 0.001) where “X” was the fold-change value and 0.001 in the equation accounted for the genes that showed no expression in certain tissues or developmental time-points. Finally heatmap was created in R-3.5.3 using pheatmap-package v.1.0.12 [54]

### Fruit growth measurements

Measurements were taken from fruit harvested for gene expression and microscopy analysis (see below). Samples were collected in four replicates, every replicate consisted of multiple fruit: 14 from 7 DAA, 8 from 14 to 28 DAA, and three from ripe fruit. Fruit diameter, height and weight were measured using digital micrometers and scale, respectively. Relative fruit growth was calculated using the formula: $x=\frac{\ln (w2)-\ln (w1)}{t2-t1}$ where w_2_ = final fruit weight, w_1_ = initial fruit weight, t_2_ = final time in days and t_1_ = initial time in days.

### Microscopy analyses

For microscopy analyses, for each replicate, two flowers and two fruit from each of the developmental stages described above (7–28 DAA) and ripe fruit were used. A total of four replicates were used for each stage of sample collected. Flower and fruit tissue at various developmental stages were harvested into a fixative solution consisting of chromic acid (0.3%), acetic acid (2%) and formaldehyde (10%) (CRAF III) for subsequent microscope analysis. For flower and fruit at 7 DAA, fixed tissues were embedded in the optimum cutting temperature (OCT) compound and sections around 20–30 μm of thickness were prepared using a cryostat microtome (Leica Jung Frigocut 2800 N). Fruit at 14 DAA, 21 DAA and 28 DAA were first cut into ~ 0.5 cm^2^ sections. Subsequently, sections of around 60 to 120 μm thickness were obtained using a Vibratome 3000 Plus Sectioning System (TED PELLA, INC, Redding, CA, USA). All the sections were stained with 0.1% toluidine blue and images were captured with a BX51 microscope (Olympus, Shinjuku, Tokio, Japan). The images were analyzed using ImageJ software to measure average cell area from 30 cells per section. Relative cell expansion rate was calculated with the formula: $x=\frac{\ln (a2)-\ln (a1)}{t2-t1}$ where a_2_ = final cell area, a_1_ = initial cell area, t_2_ = final time in days and t_1_ = initial time in days.

### Statistical and correlation analysis

Statistical analysis (one-way analysis of variance for a completely randomized design) was performed using JMP Pro 14 (SAS Institute, Cary, NC, USA). Means were separated using Tukey’s honestly significant difference (HSD) test (α = 0.05). Correlations between cell expansion rate and *EXP* gene transcript abundance in fruit were determined using pairwise correlations using JMP Pro 14 (SAS Institute, Cary, NC, USA).

## Supplementary information

**Additional file 1: Table 1.** Primers for *CaEXPAs* and reference genes.

**Additional file 2: Figure 1.** Alignment of all EXP protein sequences from *C. annuum* and representative sequences from Arabidopsis for each family, EXPA, EXPB, EXPLA, and EXPLB. All conserved amino acids are shown above the alignments for each family based on [21].

**Additional file 3: Figure 2.** Relative transcript abundance of 10 *CaEXPA* genes in flower and fruit development. These 10 *CaEXPA* genes displayed relatively lower transcript abundance during fruit development in Fig. 3. Values are means and standard errors of at least three replicates. Means separation was performed using Tukey’s HSD following test of significance using ANOVA (α = 0.05). Means followed by a different letter are significantly different.

**Additional file 4: Figure 3.** Phylogenetic analyses of EXPANSINS in bell pepper, tomato, Arabidopsis and rice. Phylogenetic tree performed using the neighbor joining method in MEGA 7.0 with 1000 bootstraps with p-distance model. Tomato sequences were obtained from [12], Arabidopsis from TAIR (https://www.arabidopsis.org/browse/genefamily/expansin.jsp) [14]; and rice (http://personal.psu.edu/fsl/ExpCentral/other_species.htm#rice%20sequences) [18].

## Data Availability

Datasets used in the current study are available from the corresponding author on reasonable request.

## Abbreviations

*18S rRNA*: *18S RIBOSOMAL RNA*
*C. annuum*: *Capsicum annuum*
CBM63: carbohydrate binding module family 63
CRAF III: chromic acid (0.3%), acetic acid (2%) and formaldehyde (10%)
DAA: days after anthesis
DEPC: diethyl pyrocarbonate
DPBB: double-psi beta-barrel
EXPs: Expansins
*EXPLA*: *EXPANSIN A-like*
*EXPLB*: *EXPANSIN B-like*
*GAPD*: *GLYCERALDEHYDE-3-PHOSPHATE DEHYDROGENASE*
GH45: glycosyl hydrolase family 45
NCBI: National Center for Biotechnology Information
OCT: optimum cutting temperature
*UBE2*: *UBIQUITIN-CONJUGATING ENZYME*
*EXPA*: *α- EXPANSIN*
*EXPB*: *β- EXPANSIN*
