# Supplementary material for: Temporal expression patterns of fruit-specific α- EXPANSINS during cell expansion in bell pepper (Capsicum annuum L.)
Source: BMC Plant Biol. 2020 May 28;20:241. doi: 10.1186/s12870-020-02452-x (PMC7254744; doi:10.1186/s12870-020-02452-x)
Supplement: Supplementary file 1 — Additional file 1: Table 1. Primers for CaEXPAs and reference genes. [file 12870_2020_2452_MOESM1_ESM.docx]

| **Additional Table 1. Primers for CaEXPAs and reference genes** | | | |
| --- | --- | --- | --- |
| **Gene** | | **Primers** | **Amplicon (bp)** |
| *CaEXPA1* | F 5’ CCAAGCATTTGCAACTCTTTCAGGCC 3’ | | 192 |
|  | R 5’ GACCCGACTAATCCGCTTTAGCCTATG 3’ | |  |
| *CaEXPA2* | F 5’ GGTGCAATCCAGGCCAAGTCATAAC 3’ | | 156 |
|  | R 5’ CAACTCCGGCTTTGTACTTGGCAATC 3’ | |  |
| *CaEXPA3* | F 5’ GCAATGCAGTGGTGGACTCTTTCAC 3’ | | 171 |
|  | R 5’ CCACATGTCCGTTGCTCATTACTGC 3’ | |  |
| *CaEXPA4* | F 5’ GATCACAAATGTGGCCGGTGCAG 3’ | | 174 |
|  | R 5’ GAAGTAGAAGTGCGATGGTCACTGCC 3’ | |  |
| *CaEXPA5* | F 5’ GGCCAAACCTTTGAAGGCAAGCAG 3’ | | 184 |
|  | R 5’ CAACACAATCGCAGAGCTTTGCTCAG 3’ | |  |
| *CaEXPA6* | F 5’ CAAGGGTTCAAGAACTCAGTGGCAAC 3’ | | 164 |
|  | R 5’ GGCCAAAGGACCAAGAACTAGGAGC 3’ | |  |
| *CaEXPA7* | F 5’ GTGGGAGGTTCAGGAGATGTCAATGC 3’ | | 155 |
|  | R 5’ GTGCGGCCATCACCTGTAGTAACC 3’ | |  |
| *CaEXPA8* | F 5’ CTCTTCATGGTTACCCATGCAGCG 3’ | | 152 |
|  | R 5’ GACCAACTTGCCAGTTAGCAGGAAC 3’ | |  |
| *CaEXPA9* | F 5’ GGTGGCATTGTACCTGTCCTCTACC 3’ | | 214 |
|  | R 5’ GACGCGCATTGGATTGCCAATTGG 3’ | |  |
| *CaEXPA10* | F 5’ CGCGATCGAGTAGTGGAACTTGAAGC 3’ | | 174 |
|  | R 5’ GTCAGCAACACTGGTCTATGTGCAAC 3’ | |  |
| *CaEXPA11* | F 5’ GGTAATAATGGAGGGTGGCAAAGTGC 3’ | | 183 |
|  | R 5’ GAGCTGGTAACAAGCACCACATGTC 3’ | |  |
| *CaEXPA12* | F 5’ GCCTCAAAGGTTCAAAGACTGGTTGG 3’ | | 158 |
|  | R 5’ CCAGTGTGCAGGTGCAATATTCCATG 3’ | |  |
| *CaEXPA13* | F 5’ CACCAGCTAACTGGCGATTTGGAC 3’ | | 137 |
|  | R 5’ CACAAACTGACAGGTGAAAGCCTCTC 3’ | |  |
| *CaEXPA14* | F 5’ CCATCCCACTGGCAATTTGGTCAGAC 3’ | | 186 |
|  | R 5’ GCCGCTTCAGCTCATATACGTACACC 3’ | |  |
| *CaEXPA15* | F 5’ GCTGTGGCGCTTGTTTCGAGATTAAG 3’ | | 182 |
|  | R 5’ GAGGAACATAGGCATGGCAAGGTC 3’ | |  |
| *CaEXPA16* | F 5’ GGACAAGCATGTGGTGCTTGCTACAG 3’ | | 180 |
|  | R 5’ GCGAAGGAAAGCAGGCATGGACATG 3’ | |  |
| *CaEXPA17* | F 5’ CATACCCATTACCACTGACCAGAGGC 3’ | | 183 |
|  | R 5’ CCGGTAACCCATAACTTTCGTCCGG 3’ | |  |
| *CaEXPA18* | F 5’ GCTGGCATCGTCCCTGTTACCTATC 3’ | | 121 |
|  | R 5’ CAGCACCTGCCACGTTGGTAACTATG 3’ | |  |
| *CaEXPA19* | F 5’ GATCGAGAACTGGTTGGCAACCAATG 3’ | | 156 |
|  | R 5’ CCAAATGACCAATGAGCAGGAGCAAC 3’ | |  |
| *CaEXPA20* | F 5’ CAGCAGATTCTGGATGGACAAGTGC 3’ | | 173 |
|  | R 5’ GCATTGGCCACATGATCCTCCATC 3’ | |  |
| *CaEXPA21* | F 5’ GTTCCTGTTTCGTATCGCAGGGTACC 3’ | | 218 |
|  | R 5’ GCTTGGCCACTTAGTGTTGCAAATGC 3’ | |  |
| *Ubiquitin-conjugating enzyme* | F 5’ CACGACGCAGCTGCTGTATTGAGAG 3’ | | 223 |
|  | R 5’ CCGAATGCGTAGCAGAAAGACCACTG 3’ | |  |
| *Glyceraldehyde-3-phosphate dehydrogenase* | F 5’ CAGTTCCGAGCACGATGTTGCCTTAG 3’ | | 132 |
|  | R 5’ CCAACAGTAAACAGACGGCCACACTC 3’ | |  |
| *18S ribosomal RNA* | F 5’ CGGGTGACGGAGAATTAGGGTTCG 3’ | | 156 |
|  | R 5’ CCAATTACCAGACTCATTGAGCCCGG 3’ | |  |
