# Supplementary material for: Temporal expression patterns of fruit-specific α- EXPANSINS during cell expansion in bell pepper (Capsicum annuum L.)
Source: BMC Plant Biol. 2020 May 28;20:241. doi: 10.1186/s12870-020-02452-x (PMC7254744; doi:10.1186/s12870-020-02452-x)
Supplement: Supplementary file 2 — Additional file 2: Figure 1. Alignment of all EXP protein sequences from C. annuum and representative sequences from Arabidopsis for each family, EXPA, EXPB, EXPLA, and EXPLB. All conserved amino acids are shown above the alignments for each family based on [21]. [file 12870_2020_2452_MOESM2_ESM.pdf]

```
AtEXPA2     1 --------------------MNLTE----------YSHILFLSL-CT-LN--FC--L---
CaEXPA1     1 -----------------------------------MASFNSKWSLICFLI--ICSM-TFF
CaEXPA2     1 -----------------------------------MAKRQSFFILFITVV--VIFLFLAF
CaEXPA3     1 --------------------------------------MAPLVI-FL-VC--LLAIA---
CaEXPA4     1 MKICVHLFSLFSPASISSTAFPFIFFNLKRVNPQLQLSMGIIIT-FILAL--VLLGSLV-
CaEXPA5     1 -----------------------------------MLLLLSLLVAF---T--LFSPT-PF
CaEXPA6     1 --------------------------------------MALLGL-LL-MG--ISLMF---
CaEXPA7     1 --------------------------------------MSILWF-SI-IG--LICIF---
CaEXPA8     1 -----------------------------------MRIYQSFFILFITIV--VSCLVTTF
CaEXPA9     1 -----------------------------------MAKICILALGF-IFG--LFNIL---
CaEXPA10    1 ------------------------------------------MLLQ---L--LLTVL-PL
CaEXPA11    1 ----------------MAVYYPL-------------SAFSILSL-IF-LI--FLSLC---
CaEXPA12    1 --------------------------------------MGVLNVACVALL--FSLILVA-
CaEXPA13    1 ------------------MDVPATF----------FTHFAFFFL-LL-LS--LLSFC---
CaEXPA14    1 --------------------------------------MEILK--LSLII--ISLVIVG-
CaEXPA15    1 --------------------------------------MAVNWTLCI-AT--LVCFVTA-
CaEXPA16    1 ------------------------------MEKKPVPRISILGF--V-LL--FTCSN---
CaEXPA17    1 --------------------------------------MAVTTMLCIITT--LICFLTD-
CaEXPA18    1 --------------------------------------MSITWMLICIAS--LVSFLTS-
CaEXPA19    1 --------------------------------------MAYFGI-CF-VG--LLAMV---
CaEXPA20    1 -----------------------------------MANYGILALGY-IIG--ICTLF---
CaEXPA21    1 -----------------------------------MAPFHHRWTLTIFLI--VVAIISLV

AtEXPB1     1 ------------------------------------------MQLFPVILPTLCVFLHLL
CaEXPB1     1 ---------------MMSTIMAL-------------NPFTVFTYLTIFSL--LT------
CaEXPB2     1 --------------------MAA-------------NNLS--KCFAIWMI--IV------
CaEXPB3     1 --------------------MAA-------------NNLS--TYFSLWII--IA------
CaEXPB4     1 ---------------------MT-------------SNFP--QFLFVYLV--LD------
CaEXPB5     1 ----------------MGTH----------------SPKFTFTNIFYFVVATVVFF-EPF
CaEXPB6     1 ----------------MD-Y----------------STYVVLVVLFFFVVVD--------
CaEXPB7     1 ---------------------MS-------------FNL---PFIFTFLA--LC------

AtEXLA1     1 -----------------------------------------MGS-FLFLI--VV--IF--
CaEXLA1     1 ---------------------------------------------MALLF--LV--FL--

AtEXLB1     1 ------------------------M----------KHSHVLLLL-FVQVI--VL--LP--
CaEXLB1     1 --------------------MIC-Q----------IEKCSI--L-ALCFL--IL--LP--
CaEXLB2     1 --------------------MD--F----------SLKHCT--L-LICLI--LI--LP--
CaEXLB3     1 --------------------MAI-F----------LNNFST--F-MICMI--LV--LP--
CaEXLB4     1 --------------------MD--E----------SLKYSS--I-FMCMI--ML--LP--
CaEXLB5     1 ---------------------MV-F----------TNSHCIFL---LVTL--II--CP--
CaEXLB6     1 --------------------MA---------------SSQILAI-FSTFL--IF--MQ--
CaEXLB7     1 ---------------------M--F----------FLKNNY--T-FLFIV--LL--LP--
CaEXLB8     1 ---------------------M--V----------FLQYNY--A-FLFIV--ML--LP--
CaEXLB9     1 --------------------MA---------------PLQFLSV-FVASF--LF--ML-
```

```
                                    W                    C
AtEXPA2     22 -----------YSINSDDNGGWERGHATFYGGADASGTMGGACGYGNLHS-QGYGLQTAA
CaEXPA1     23 HQAM---AAYYSTPLTFKAMPWKQAYATFYGDESASETMGGACGYGNLFN-SGYGTATAA
CaEXPA2     24 ESK--------EVEGGLNGNGWRNARATFYGDMKGGETMQGACGYGNLFQ-QGYGLETAA
CaEXPA3     16 -----------SFVEGNRKGEWINGHATFYGGGDASGTMGGACGYGNLYE-QGYGTRTAA
CaEXPA4     57 VEA--------RISGVYSGGSWQTAHATFYGGSDASGTMGGACGYGNLYS-QGYGVNTAA
CaEXPA5     20 VHSHYNWSPSSSSTSSNFQSEWRPARATYYAAADPRDVVGGACGYGDLER-NGYGKATAG
CaEXPA6     16 -----------QSVHGY--GGWINAHATFYGGGDASGTMGGACGYGNLYS-SGYGTNTAA
CaEXPA7     16 -----------INCVHGNEGGWIEAHATFYGGGDASGTMGGACGYGNLYS-EGYGTNTAA
CaEXPA8     24 ESK--------ELE------GWKHGRATFYGDMKGGETMQGACGYGDLFK-QGYGLKTAA
CaEXPA9     20 --L--------SANAFSA-SGWMSAHATFYGGSDASGTMGGACGYGNLYS-TGYGTNTAA
CaEXPA10    13 VHSHYNWSPTAAAT---QKFEWRPARATYYAPVDPRDAVGGACGYGDLER-SGYGKSTAG
CaEXPA11    25 -----------INGSFGNNGGWQSAHATLYGGGDASGTMGGACGYGNLYT-QGYGTYTAA
CaEXPA12    20 AEA--------RIPGVYNGGAWQSAHATFYGGSDASGTMGGACGYGNLYS-QGYGANNGA
CaEXPA13    26 -----------FHGSFADYGDWQSAHATFYGGSDASGTMGGACGYGNLYS-QGYGTNTAA
CaEXPA14    18 VEG--------RIPGVYRGGSWQTAHATFYGGSDASGTMGGACGYGNLYS-QGYGVNNAA
CaEXPA15    19 VTA--------KIPGVYTGGPWTSAHATFYGGSDASGTMGGACGYGNLYS-QGYGVNNAA
CaEXPA16    23 -----------LFIEEINAQNWLNGHATFYGVNQDPSTFGGACGYDNPYH-AGFGVHTTA
CaEXPA17    20 VNA--------RIPGVYTGGPWQDAHATFYGGSDASGTMGGACGYGNLYS-QGYGVNNAA
CaEXPA18    20 VDA--------KVRGVYTVGPWQDAHATFYGGSDASGTMGGACGYGNLYS-TGYGVNNAA
CaEXPA19    16 -----------STVYGYGGGGWINAHATFYGGGDASGTMGGACGYGNLYS-QGYGTNTAA
CaEXPA20    20 --S--------SANGFSADSGWTSAHATFYGGADASGTMGGACGYGNLYS-TGYGTRTAA
CaEXPA21    24 DRTK---AGGYGTPSGFQATPWKLAHATFYGDETASATMGGACGYGNLIS-SGYGTDTVA

                                    W      W             C
AtEXPB1     19 ISGSGSTPPLTHSNQQVAATRWLPATATWYGSAEGDGSSGGACGYGSLVDVKPFKARVGA
CaEXPB1     25 ISCSCFNPKILNVSKTESDSNWSPAGATWYGSPNGAGSDGGACGYTNAVDQPPFSSLIAA
CaEXPB2     18 VFC---S--CLFTFSSCQPSGFSPAVATYYTNPTSPGSGG-ACGLENDVVGAPYNAMITA
CaEXPB3     18 FFG---S--CLVTFSAFQPSGFSPAVATYYTNPTSPGSGG-ACGLENDVVSSPYNAMITA
CaEXPB4     17 LVSVCSS--RSFITGNFNTSGFSSAVATWYGDPTGAGSGG-ACGLEDDVAKAPYNAMITA
CaEXPB5     28 M-------AFAGPLRRVVDTHWYPATATWYGSPEGDGSTGGACGYGSMVDVRPFRARVGA
CaEXPB6     20 V-------ALAGP-KGAGNPHWHSASATWYGSPNGDGSDGGACGYGTMVDVKPYKARVGA
CaEXPB7     16 LFQYCTC--AHFKSLNASLAGFQPAVATFYGSPTGAGSDGVACGYTNAVSQSPFNSFVSA

                                C  C                     C
AtEXLA1     13 ---------LFSSSVNACDRCLHRSKAAYFSSASA--LSSGACAYGSMAT-SFFAGHIAA
CaEXLA1     10 ---------LFVSSVTACDRCVHRSKVAYFSSASS--LLSGACGYGDLAT-GFNGGRLAA

                                                         C
AtEXLB1     20 ---------LLCLSDD-----FVNSRATYYGSPDCKANPRGHCGYGEFGR-DINNGEVSG
CaEXLB1     21 ---------ILCSCDGIGVDTSVTAKASFYGSIGHLVKPSGACGYGEYGR-TVNSGAFCG
CaEXLB2     20 ---------ALCYSQTY------VSKAGYYNTDDGMGNPKGACGYEEYGR-TVNNGEVCA
CaEXLB3     21 ---------AFCYGTEY-----YDSKATYYKTPDGKGTPTGACGYGQHGK-YVNDGLVTG
CaEXLB4     20 ---------AFSYSQTL------VSKASFYSTSDGMGTPSGACGYGDYGK-DVNNGEVCT
CaEXLB5     20 ---------AICYSQEA---NSLYTRATYYGSPDCYGTPSGACGFGEYGR-TIYDGKVSG
CaEXLB6     19 ---------IYFGN----AQSFIQSRAAYYPNSEEKGTESGACGFGTFGA-TINGGDVSA
CaEXLB7     19 ---------MLCYSNNY------VSRVSYYTTTDGMGTPSGACGYGDYGK-DVNNGDVCT
CaEXLB8     19 ---------MLCYSSNY------VPKASYYSTPDGMGTSSGACGYGVYGK-DVNNGDVCT
CaEXLB9     19 ---------T-LGNSQTCPDCFTRSRAGHYPNSEEKGTETGSCGFGTFGA-TVNGGDVSA
```

```
                             C  C    C         C              C            C
AtEXPA2     70 LSTALFNSGQKCGACFELQCED-DPE--WCIPG--SIIVSATNFCPPNFALANDNGGWCN
CaEXPA1     79 LSTVLFSNGYSCGQCFQIMCVK--S--KFCYKG--YTTITATNLCPPNWSQDSNHGGWCN
CaEXPA2     75 LSTALFNKGSTCGACFQLMCVN-VPK--WCNPGQ-VITITATNFCPPDYSKTV--DIWCN
CaEXPA3     64 LSTTLFNNGLSCGSCYEIKCIN-EHK--WCLSD--SIRVTATNLCPP--------GGWCN
CaEXPA4    108 LSTALFNNGLSCGACFEIKCRN-DPHWKWCLPGSPSILITATNFCPPNYALSNDNGGWCN
CaEXPA5     79 LSTVLFDKGQICGACFEVRCVE-DLR--WCIPGT-SIIVTATNFCAPNYGFDLDGGGHCN
CaEXPA6     62 LSTALFNNGLSCGQCFQLMCVN-ARQ--YCLPG--IITVTATNFCPP--------GGWCD
CaEXPA7     64 LSTALFNNGLSCGACFELKCVG-DSK--WCLPG--SIVVTATNFCPPNFALPNNAGGWCN
CaEXPA8     69 LSTALFNNGATCGACFQLMCVD-APQ--SCHLGNRIITITATNFCPPDYSKTE--DIWCN
CaEXPA9     68 LSTALFNDGASCGQCYKIMCDYN-QDSKWCIKGT-SITITATNFCPPNYALPSNNGGWCN
CaEXPA10    69 LSTVLFEKGQICGACFEVRCVE-ELK--WCIPGT-SIIVTATNFCAPNYGLESDGGGHCN
CaEXPA11    73 ISTALFNNGLTCGACYQLKCNNNDGT--LCLPG--TITVTATNFCPQNPSLPSNNGGWCN
CaEXPA12    71 LSTALFNNGLSCGACFEIKCDN-YPQ--WCHPGSPSIFITATNFCPPNFALPNDNGGWCN
CaEXPA13    74 LSTELFNNGLACGSCYELTCSN-DAP--WCLPG--TISVTATNFCPQNPSEPNDNGGWCN
CaEXPA14    69 LSTALFNNGLSCGACFEIKCDN-EPQ--WCHPGSPSILVTATNFCPPNYALPNDNGGWCN
CaEXPA15    70 LSTALFNNGLSCGACFEIKCTN-AKE--YCNPGNPSIFVTATNFCPPNYALPNDNGGWCN
CaEXPA16    71 LSSALFRNGQACGACYRVRCNR-QLDRRWCLPHG-AVTVTATNFCPPN-----NHGGWCD
CaEXPA17    71 LSTVLFNSGLSCGACFELKCVN-DGK--WCLPGNPSIFVTATNFCPPNFALPNDDGGWCN
CaEXPA18    71 LSTALFNSGLSCGACFEIKCTD--PK--WCTPGNPSILVTGTNFCPPNYALPNDNGGWCN
CaEXPA19    64 LSTAMFNNGLSCGSCFELRCVN-DGQ--GCLPG--SIVVTATNFCPPNNALPNNAGGWCN
CaEXPA20    69 LSTALFNDGGSCGQCYKIICDYK-AGPQWCKKGV-SVTITATNFCPPNYNLPSNNGGWCN
CaEXPA21    80 LSSVLYNKGCACGQCYQIKCVQ--S--SSCYST--TVTVTATNLCPPNPSQDSNNGGWCN

                             C  C    C         C
AtEXPB1     79 VSPILFKGGEGCGACYKVRCLD----KTICSKR--AVTIIATDQSP---------SGPSA
CaEXPB1     85 AGPSLFKSGKGCGACFQVKCTE----NKACSGK--PVRVVITDSCP---------GGPCL
CaEXPB2     72 GNQDLFKQGSGCGACYQVMCTQ--TQNSHCSGN--PITVTLTDECP---------G-ACN
CaEXPB3     72 GNQALFKQSSGCGACYQVMCTQ--FHNPHCSGS--PITVTLTDEYP---------G-ACN
CaEXPB4     74 GNQVLFQHGSGCGACYQVMCNQ----NKECSGK--PITVALTDECP---------G-TCN
CaEXPB5     81 VSPILFKNGEGCGACYKVKCLD----SSICSRR--AATVIITDECP---------GGYCS
CaEXPB6     72 VSSALFKSGEGCGACYKVKCLD----KSICSKR--AVTVIVTDESP---------A--LT
CaEXPB7     74 GNGPLFRKGLGCGACYQVICNL-----APCSGK--PVTVTITDECP---------S-C--

                             C  C    C         C
AtEXLA1     61 AIPSIYKDGAGCGACFQVRCKN----PKLCSTK--GTIVMITDLNK---------S----
CaEXLA1     58 AVPSIYKEGAGCGACYQMRCKD----PKICSKA--GTTVIVTDLNT---------N----

                             C  C    C         C
AtEXLB1     65 VSWRLWNNGTGCGACYQVRCKI----PPHCSEE--GVYVVATDSGE---------G----
CaEXLB1     71 VS-KLYSNGTSCGACYHVKCKH----PEYCNEE--GTDVVVTDSGE---------E----
CaEXLB2     64 VSRRLFKNGASCGGCYQVRCRN----KGLCSQE--GVKVMATDYGE---------G----
CaEXLB3     66 VSWKLYKNGAGCGACYQVRCKD----KSLCSDK--GVKVMVTDSGE---------G----
CaEXLB4     64 ASRRIYRNGAGCGACYQVRCKN----TALCSAE--GTKVVVTDSAD---------G----
CaEXLB5     67 VS-GLYKNGTGCGACYQVRCKV----PSHCTDE--GTKIVVTDYGE---------G----
CaEXLB6     65 AS-DLYRDGLGCGACYMVRCTN----SNYCSEN--GVNVVITDHGA---------S----
CaEXLB7     63 ASKRLYKNGAACGACYQVRCKD----KELCSEE--GAKVVVTDNGE---------G----
CaEXLB8     63 ASRRLYKNGAACGACYQVRCKD----KELCSEE--GAKVVLTDSGE---------A----
CaEXLB9     68 AS-ELYRNGLGCGACYQVRCID----SNYCSDK--GVTVVVTDQGA---------G----
```

```
                   HFD                                      C
AtEXPA2    125 P-PLKHFDLAEPAFLQIAQ--Y-R-------AGIVPVAF----RRVPCEKGG--GIRFTIN
CaEXPA1    133 P-PRQHFDMSKPAFMKIAQ--W-K-------AGIVPVAY----RRVPCVKKG--GIKFALQ
CaEXPA2    129 P-PQRHFDLSMPMFLKIAK--Y-K-------AGVVPVVY----KRVNCHKKG--GLKFEIK
CaEXPA3    111 P-SLHHFDLSQPIFQHIAQ--Y-R-------AGVVPIAY----RRVPCRRKG--GIRFTIN
CaEXPA4    167 P-PRSHFDLAMPMFLKIAQ--Y-R-------AGIVPVTY----RRIPCRKQG--GIRFTMN
CaEXPA5    135 P-PNAHFVLPIEAFEKIAI--W-K-------ASNMPIQY----RRIKCRKEG--GVRFTIG
CaEXPA6    109 P-PNHHFDLSQPIFLRIAQ--Y-R-------AGIVPVAY----RRVPCRRRG--GIRFTIN
CaEXPA7    119 P-PLHHFDLSQPVFQQMAQ--Y-R-------AGIVPVAY----RRVPCQKRG--GIRFTMN
CaEXPA8    124 P-PQEHFDLSMPMFLEIAK--Y-E-------AGVVPVKY----RRVHCQKTG--GLKFEMK
CaEXPA9    126 P-PRPHFDMAQPAWEHIGI--Y-R-------GGIVPVLY----QRIPCEKKG--GVRFTIN
CaEXPA10   125 V-PNAHFVLPIEAFEKIAI--W-K-------ASNMPVQY----RRIKCRKEG--GVRFTIN
CaEXPA11   129 A-PLQHFDLAQPAFLKIAQ--Y-K-------AGIIPVSF----RRVPCIKKG--GIRFTIN
CaEXPA12   128 P-PRPHFDLAMPMFLHIAE--Y-R-------AGIVPVVY----RRVPCRKKG--GIRFTIN
CaEXPA13   129 P-PRQHFDLAEPAFLQIAQ--Y-R-------AGIVPVSF----RRVPCSKKG--GIRFTIN
CaEXPA14   126 P-PRSHFDLAMPMFLKIAE--Y-R-------AGIVPVVY----RRVPCRKQG--GIRFTIN
CaEXPA15   127 P-PRPHFDLAMPMFLKIAV--Y-R-------AGIVPVNY----RRVPCRKQG--GIRFTVN
CaEXPA16   124 A-PRQHFDMSMPAFLRIAR--QGN------EGVVPVLY----TRVTCRRKG--GVRFTLK
CaEXPA17   128 P-PRSHFDLAMPMFLKIGQ--Y-R-------AGIVPVTY----RRVPCRKQG--GIRFTIN
CaEXPA18   127 P-PRPHFDLAMPMFLKIAE--Y-R-------AGIVPVTY----RRVPCKKQG--GIRFTIN
CaEXPA19   119 P-PLHHFDLSQPIFQHIAH--Y-K-------AGIVPVAY----RRVPCRRRG--GIRFTIN
CaEXPA20   127 P-PRPHFDMAQPAWEKIGI--Y-K-------GGIVPVLY----KRVPCKKHG--GVKFTIN
CaEXPA21   134 P-PRTHFDMAKPAFMKIAQ--W-K-------AGIVPVSY----RRVPCVKKG--GVKLSFQ

                   HFD                                      C
AtEXPB1    124 KAKHTHFDLSGAAFGHMAI--PGHNGVIRNRGLLNILY----RRTACKYRG-KNIAFHVN
CaEXPB1    130 S-ESAHFDLSGTSFGAMAV--SGQADQLRNAGVLQIQY----KRVECNYPG-VKLTFHVD
CaEXPB2    118 N-DPVHFDLSGIAYGKLAK--SGEEE--------FQFFIKGDDQVACNYNS--NILFKVD
CaEXPB3    118 N-NPVHLDLSGITFGKLAK--HGAAPQLHFAGRIPIFFIKGDNQVACNDNS--NILFKVD
CaEXPB4    118 D-DPVHFDLSGTAFGAMSK--SGQADRLRKLGRIDISY----KRVTCDYKQ--NIMFKVD
CaEXPB5    126 G-GRVHFDLSGAAFGHMAV--SGYGGSLRDRGVISVIY----RRTPCKYPG-KNIAFHVN
CaEXPB6    115 K-GLVHFDLSGSAFGRMAV--SGRSTNLRNRGKISVIY----RKTPCKYPG-QNIAFHVN
CaEXPB7    115 S-GAVHFDLSGTAMGALAK--PGQADALRNLGNVAISY----QRVPCQYKT--KIAFKVD

                                                            C
AtEXLA1    102 --NQTDLVLSSRAFRAMAKPIVGADKDLLKQGIVDIEY----QRVPCDYGN-KNMNVRVE
CaEXLA1     99 --NQTDFVISSRAFMAMA--IQGKAQDVLKLGIADVEY----KRVPCDYKG-KNLAIRVE

                                                            C
AtEXLB1    106 --DGTDFILSPKAYGRMAR--PGTENQLYSFGVVNVEY----QRIPCRYAG-YNLVYKIH
CaEXLB1    111 ---NAEFILSFSAYAKLAK--PLMVAKLVAEGEVDIEY----QRVPCKYATGAHLMVKVH
CaEXLB2    105 --HGTDFILSYRGFAKLAKQ-SSMAQVLFAKGTVDVEY----RRVSCRFG--SNLMVKIH
CaEXLB3    107 --PGTDFILSSEAFAKMAKH-PKLAHMLFPKGVVDVDY----KRVSCKYG---NLKIKIN
CaEXLB4    105 --QETDLILSFKAYAKLAKN-PNLTPKLCGKGVVDIEY----RRVSCGYG--GNLMVKIH
CaEXLB5    107 --DHTDFILSVRAYTEMAT--PGMANHLLAYGVVDVEY----RRIPCRYYG-YNLMIKVH
CaEXLB6    105 --ANTDFILSQRAFSHMAQT-KDAATSLLSLGNVGIEY----TRISCSYPN-KNITFKID
CaEXLB7    104 --HGTDFILSQRAYAKLAKQ-PNKAQHLFAKGVVEVEY----RRVSCKYGG-GNLMVKVQ
CaEXLB8    104 --HGTDFILTHRAYAKLAKQ-PYMAQHLFAKGVVEVEY----RRVSCKYG--GNLMVKVH
CaEXLB9    108 --DRTDFILSKRAFARMAQT-TDAAASILPLGVVDIEY----RRVSCTYPD-KNITIKIE
```

```
                                                    W        W   W
AtEXPA2    169 --G-NPYFDLVLITNVGGAGDIRAVSLKGSKT-D--QWQSMSRNWGQNWQSNTYLR--GQ
CaEXPA1    177 --G-NGYWLLVYVMNVAGGGDVASMWVKGSKT----GWMSMSHNWGASYQAFATLS--GQ
CaEXPA2    173 --G-NPYWILVLVFNVGGVGDVVNVKIKGSKT----GWLQMGRNWGQNWQTSMQLA--GQ
CaEXPA3    155 --G-HSYFNLVLVTNVGGAGDVHAVAIKGSKT----QWQSMPRNWGQNWQSNSLLN--GQ
CaEXPA4    211 --G-FRYFNLVLITNVAGAGDIIKVWVKGSKT----NWIPLSRNWGQNWQSNAVLT--GQ
CaEXPA5    179 --G-AGIFLSVLISNVAGSGDIVAAKVKGSRT----GWLPMGRNWGQNWHVNADLK--NQ
CaEXPA6    153 --G-HSYFNLVLVTNVGGSGDVHSVYIKGSRT----QWQPMSRNWGQNWQNNAYLN--GQ
CaEXPA7    163 --G-HSYFNLVLVTNVGGSGDVNAVSVKGSRS----GWMPMSRNWGQNWQSNSFLD--GQ
CaEXPA8    168 --G-NPNWILVLVHNVGGVGDVVNVKVKGSNS----SWLPMQRNWGQNWQTHERLV--GQ
CaEXPA9    170 --G-REYFELLMISNVGGAGSVQSVQIKGSRT----NWMAMSRNWGANWQSNARLD--GQ
CaEXPA10   169 --G-AGIFLSVLISNVAGAGDIVAAKVKGSRT----GWLPMGRIWGQNWHISADLK--NQ
CaEXPA11   173 --G-HSYYNLVLVTNVGGAGDVHSVSIKGSKN----GWQLMSRNWGQNWQSNSYLN--GQ
CaEXPA12   172 --G-FRYFNLVLVTNVAGAGDIVSLSLKGSKT----GWISMSRNWGQNWQTNSVLI--GQ
CaEXPA13   173 --G-NSYFNLVLVTNVGGAGDVNSVSIKGSDS----EWQTMARNWGQNWQSNSYLN--GQ
CaEXPA14   170 --G-FSYFNLILVTNVAGAGDIMKVMVKGTRT----NWITLSRNWGQNWQTNSVLV--GQ
CaEXPA15   171 --G-FKYFNLVLVTNVAGAGDIQKVYVKGTKT----QWISMNRNWGQNWQTNAMLV--GQ
CaEXPA16   169 --G-QANFNMVMISNVGGSGDIKSVSIRGSRT-K--IWLPMHRNWGVNWQSRNDLR--TQ
CaEXPA17   172 --G-FRYFNLLLVTNVAGAGDIQKVLIKGTHT----QWITMSRNWGQNWQTNSPLV--GQ
CaEXPA18   171 --G-FNYFNMIIVTNVAGAGDIQQVQVKGTNT----KWIFMSRNWGQNWQSNVVLV--GQ
CaEXPA19   163 --G-HSYFNLVLVTNVGGSGDVHTVAVKGSRT----GWQPMSRNWGQNWQNNNYLN--GQ
CaEXPA20   171 --G-RDYFELVLVTNVAGAGSVQSVQIKGSNT----NWLTMSRNWGASWQSNAYLD--GQ
CaEXPA21   178 --G-NGYWLLVYVMNVGGSGDIASVFVKGTKT----NWLPMSHNWGASYQAFATLS--GQ

                                                    W        W   W
AtEXPB1    177 AGS-TDYWLSLLIEYEDGEGDIGSMHIRQAGS-K--EWISMKHIWGANWCIVEGP--LKG
CaEXPB1    182 SGS-NSNYFAALVEYEDGDGDLSRVDLKQALD-S-ESWQPMQQSWGAVWKLNSGSS-LVA
CaEXPB2    165 KGS-NPNFFAVVSEAVNGDGDLSLVEIKTGGA-STTAWTPMKRMIGSTWNVGIQPNTQKP
CaEXPB3    173 KGS-NPNFFAVVSKAVNGDGDLSMVEIKTGGR-S-TPWTPMKRMIGATWSVGIQPNIQRP
CaEXPB4    169 KGS-NPNFFAVAIEAENGDGKLSLVEIKPTGS---NKWFPMQQMFGATWSTNIYPDKQKP
CaEXPB5    178 EGS-TPYWLSLLVEFEDGDGDVGSMRIREARS-N--QWLEMTHLWGANWAIIGGP--LQG
CaEXPB6    167 AGS-TAYWLSLLVEFEDGDGDIGSMHIRQARS-N--QWLKMTHLWGANWCIIGGP--LQG
CaEXPB7    166 QGS-NPNFLSVNVEFENADGDLNLVEFLPSGS-T--QSITANHVFGATWSSNINPSTQPA

                                                    W            W
AtEXLA1    155 EASKKPNYLEIKLLYQGGQTEVVSIDIAQVGSSP--NWGYMTRSHGAVWVTDKVPT---G
CaEXLA1    150 ESSQKPNYLAISFLYQGGQTEIVGVDAAQVGS-S--NWNFMTRNHGAIWDTSRVPS---G

AtEXLB1    157 EKSYNPHYLAILVLYVGGVNDILAVEVWQEDC-K--EWRRMRRVFGAVHDLQNPPR---G
CaEXLB1    162 ESSKFPNYLAIVLLNKGGLSDITAIEVWEESH-K--QWKSMVRIFGAVWAVSDPPK---G
CaEXLB2    156 ERSKYPNYLSLVVMNQGGASDILALEVYEEET-S--SWISMRRAYGAVFDLSNPPD---G
CaEXLB3    157 EHSNYHGYLAIFLFNNGGYADIYAIEVYDVKT-Y--KWIPMRRSYGAVFDLQNPPK---G
CaEXLB4    156 ERSKYAEYLAIVVLNQGGATDIHVVEVYEEES-Q--QWISMRKAYGAVWDLSSPPC---G
CaEXLB5    158 ENSRFCNYLAIVPIYQSGAFDVEAVEVWQADC-K--EWKGMRKAYGAVWDMPDPPK---G
CaEXLB6    157 ESSDNPYYLAFVIWYQQGKTDISAVQLCETQN-F--VCKLLDRTRGAVWTSSSPPK---G
CaEXLB7    156 EHSKYPNYLAVVVMNQGGATDILGVEVFEEET-K--EWLSMRRAYGAVFDLSNPPS---G
CaEXLB8    155 EHSKNPHYLAIVVMNQGGATDILGVEVYEEET-K--EWISMRRAYGAVFDLWSPPS---G
CaEXLB9    160 ESSNNPHYLAFVIWYQQGKSDITAVQLCETQN-F--ACKLLDRTRGAVWTTSTPPK---G
```

```
                                                W
AtEXPA2    221 SLSFQV--T-DS-DGRTVVSY-DVVPHDWQFGQTFEGGQF-------------------
CaEXPA1    228 TLSFKL--T-SYTSHETIIAY-NVAPSNWRVGMTYQANVNFH------------------
CaEXPA2    224 TLSFQV--Q-TS-DGRWVQSN-NVVPANWQFGQTFEAKNNF-------------------
CaEXPA3    206 SLSFKV--T-TS-DGRSVVSK-NVAPASWSFGKTYTGGQFH-------------------
CaEXPA4    262 SLSFRV--K-GS-DHRTSTSW-NIVPHHWQFGQTFTGKNFRI------------------
CaEXPA5    230 PLSFEL--T-SG-DGGTLTSY-NVVPKNWNFGQTFEGKQFGS------------------
CaEXPA6    204 SLSFKV--T-TG-DGRTVVSY-NAAPSSWSFGQTFSGGQFR-------------------
CaEXPA7    214 ILSFKV--T-TG-DGRTVICS-NAIPAGWSFGKTYTGAQFN-------------------
CaEXPA8    219 SLCFQV--Q-AS-DGRWVETD-YIVPANWQVGQTFEAKTNF-------------------
CaEXPA9    221 SLSFKV--T-TS-DGVTKTFL-NVASSNWQFGQTYSSSMNF-------------------
CaEXPA10   220 PISFEI--T-SS-AGVTLTSY-NVAPKNWNFGQTYQGKQFDS------------------
CaEXPA11   224 SLSFQV--T-TS-DGRTVTSN-NVAPANWQFGQTFEGAQF-------------------
CaEXPA12   223 SLSFRV--R-AS-DRRSSTSW-NIAPAHWQFGQTFIGKNFRV------------------
CaEXPA13   224 SLSFQV--T-TS-DGRTITSY-NISPANWRFGQTFEGVQF-------------------
CaEXPA14   221 SLSFRV--R-AT-DKRQSTSW-NIVPSHWQFGQTFVGKNFRV------------------
CaEXPA15   222 ALSFRV--T-AS-DKRSSASY-NIAPANWQFGQTFQGKNFRV------------------
CaEXPA16   221 TLSFRL--T-LV-DGKTMEFI-NVVPSSWKFGQTFASRRQFY------------------
CaEXPA17   223 ALSIRV--K-AS-DHRSVTTM-NVAPTNWQFGQTFQGKNFRV------------------
CaEXPA18   222 ALSFKV--K-AS-DHRYVTSY-NVAPSNWQFGQTYEGKNFRV------------------
CaEXPA19   214 TLSFKV--T-TG-DGRSLISY-NVAPAHWSFGQTYTGAQFH-------------------
CaEXPA20   222 SISFKV--T-TT-DGVTKTFL-NVVPSSWKFGQTFSSHTQF-------------------
CaEXPA21   229 ALSFKI--T-SYTSHETIIAN-NVAPSNWQVGMTYQANNNFK------------------

                                                W
AtEXPB1    231 PFSVKL--T-TLSNNKTLSAT-DVIPSNWVPKATYTSRLNFSPVL---------------
CaEXPB1    238 PFSLKL--T-GE-SGQTVVAN-GVIPAGWQPGKTYRSVVNFKV-----------------
CaEXPB2    223 PFSLRL--T-SS-TKKSVIAQ-NVIPPGWQPRSVYKSNVNFPSQL---------------
CaEXPB3    230 PFSLRL--T-SS-TRKSVIAQ-NVIPPGWHPRSVYRSNVNFPSLL---------------
CaEXPB4    225 PFSIRI--T-SQ-NKHQVQAD-NVIPVNWQSSATYNSNVNFSPQL---------------
CaEXPB5    232 PFSVKL--T-TLSTGRALSAR-DVIPGNWSPKATYTSRLNFY------------------
CaEXPB6    221 PFSIKL--T-TLSKRVTLSAR-DVIPSKWTPKATYTSRLNFLK-----------------
CaEXPB7    222 PYSIRI--T-TE-FNKKLTAT-NVIPIGWKPGQTYVSNVNF-------------------

                                       W        W                    C  C    W
AtEXLA1    210 AIQFRFVVT-GGYDGKMIWSQ-SVLPSNWEAGKIYDAGVQITDIAQEGCDPCDAHIWN--
CaEXLA1    204 ALQFRFVVT-AGYDGKWNWAK-SVLPADWKNGVIYDTGLQITDIAQEGCSPCDDGNWKLH

                                                W
AtEXLB1    211 TLTLRFLVY-GSAGINWIQSP-NAIPADWTAGATYDSNILLT------------------
CaEXLB1    216 ALKCRFQVR-GSAGVVWVQPK-KLIPSDWKAGILIDTFIQLT------------------
CaEXLB2    210 DLQVRFLIS-AAAETKWVQSERAIIPAEWSVGETIETDIQVS------------------
CaEXLB3    211 DLKLRIQIK-EGEKTKWVSSDKTVIPDYWKPGSIYETDIQIP------------------
CaEXLB4    210 DLKVRFLTS-TSVETKWVQSDKAVIPSQWKAGLTIETDIQLT------------------
CaEXLB5    212 SLTFRVQVSVNGEAAKWVQLT-DVLPDEWKAGIAYDTYLLLD------------------
CaEXLB6    211 ELQIRMLLSFDDGDEKWIIPV-NNIPENWKVGEIYDSGIQVDQ-----------------
CaEXLB7    210 ELKVRFLTS-AGAETKWVESDKAVIPAQWKAGITIETDIQLS------------------
CaEXLB8    209 DLKVRFLTS-AGTETKWVESDNAVIPAKWMAGISIETDIQLS------------------
CaEXLB9    214 PLQIRMLLSVDDGDETWVVPV-NNIPENWKAGDTYDSGIQLDA-----------------
```
