# Supplementary figures and images for: Temporal expression patterns of fruit-specific α- EXPANSINS during cell expansion in bell pepper (Capsicum annuum L.)
Source: BMC Plant Biol. 2020 May 28;20:241. doi: 10.1186/s12870-020-02452-x (PMC7254744; doi:10.1186/s12870-020-02452-x)

Relative expression

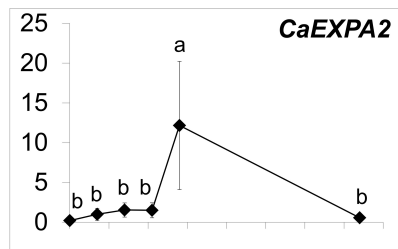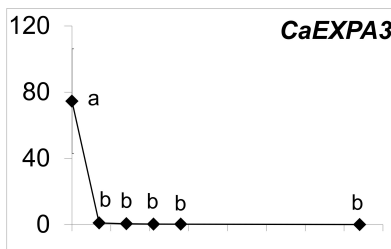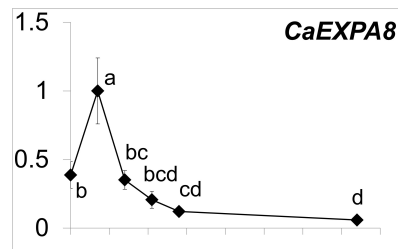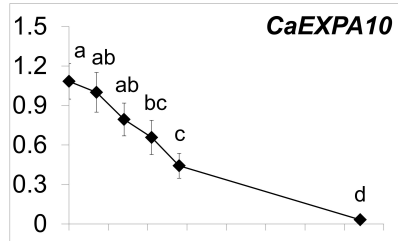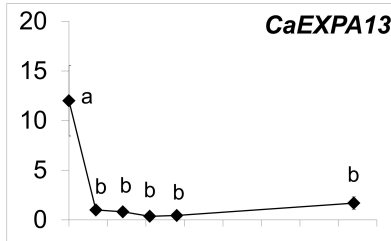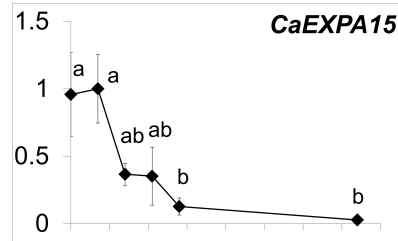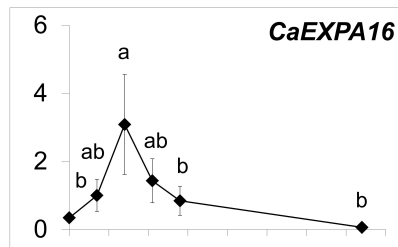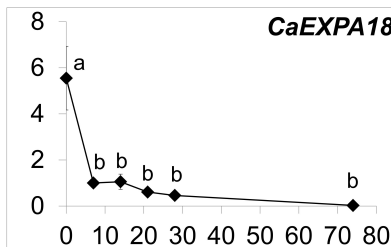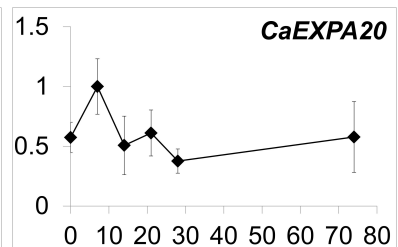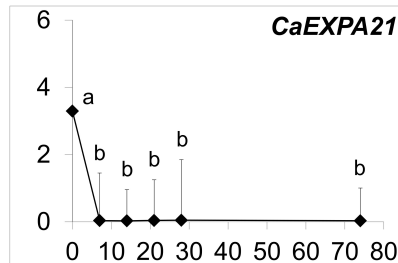

Days after anthesis

Supplement: Supplementary file 3 — Additional file 3: Figure 2. Relative transcript abundance of 10 CaEXPA genes in flower and fruit development. These 10 CaEXPA genes displayed relatively lower transcript abundance during fruit development in Fig. 3. Values are means and standard errors of at least three replicates. Means separation was performed using Tukey’s HSD following test of significance using ANOVA (α = 0.05). Means followed by a different letter are significantly different. [file 12870_2020_2452_MOESM3_ESM.pdf]

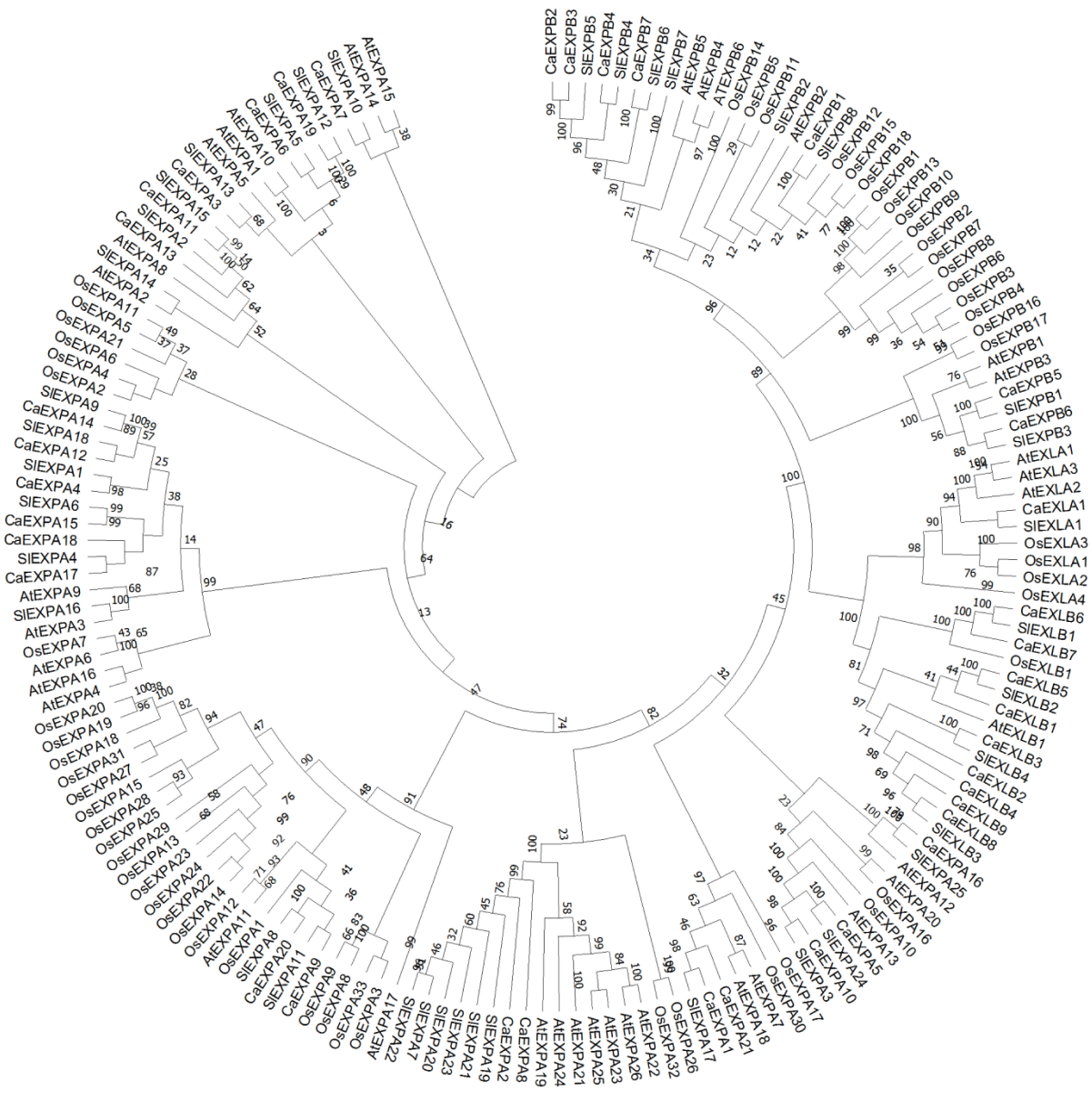

Supplement: Supplementary file 4 — Additional file 4: Figure 3. Phylogenetic analyses of EXPANSINS in bell pepper, tomato, Arabidopsis and rice. Phylogenetic tree performed using the neighbor joining method in MEGA 7.0 with 1000 bootstraps with p-distance model. Tomato sequences were obtained from [12], Arabidopsis from TAIR (https://www.arabidopsis.org/browse/genefamily/expansin.jsp) [14]; and rice (http://personal.psu.edu/fsl/ExpCentral/other_species.htm#rice%20sequences) [18]. [file 12870_2020_2452_MOESM4_ESM.pdf]
